# Publication of data collection forms from NHLBI funded sickle cell disease implementation consortium (SCDIC) registry

**DOI:** 10.1186/s13023-020-01457-x

**Published:** 2020-07-07

**Authors:** Jeffrey A. Glassberg, Elizabeth A. Linton, Katrina Burson, Tabitha Hendershot, Joseph Telfair, Julie Kanter, Victor R. Gordeuk, Allison A. King, Cathy L. Melvin, Nirmish Shah, Jane S. Hankins, Axel Yannick Epié, Lynne D. Richardson, for the Sickle Cell Disease Implementation Consortium

**Affiliations:** 1 Department of Emergency Medicine, Icahn School of Medicine at Mount Sinai, 1 Gustave L. Levy Place, Box 1620, New York, NY 10029 USA; 2 RTI International, Research Triangle Park, Durham, NC USA; 3 Jiann-Ping Hsu College of Public Health, Georgia Southern University, Statesboro, GA USA; 4 Division of Hematology & Oncology, University of Alabama at Birmingham School of Medicine, Birmingham, AL USA; 5 Division of Pediatric Hematology-Oncology, University of Illinois at Chicago, Chicago, IL USA; 6 School of Medicine, Washington University in St. Louis, St. Louis, MO USA; 7 Department of Public Health Sciences, Medical University of South Carolina, Charleston, SC USA; 8 Division of Hematology, Duke University School of Medicine, Durham, NC USA; 9 Department of Hematology, St. Jude Children’s Research Hospital, Memphis, TN USA

**Keywords:** Sickle cell disease, Standardized data collection tools, SCDIC registry forms, Accurate characterization

## Abstract

**Background:**

Sickle cell disease (SCD) is an autosomal recessive blood disorder affecting approximately 100,000 Americans and 3.1 million people globally. The scarcity of relevant knowledge and experience with rare diseases creates a unique need for cooperation and infrastructure to overcome challenges in translating basic research advances into clinical advances. Despite registry initiatives in SCD, the unavailability of descriptions of the selection process and copies of final data collection tools, coupled with incomplete representation of the SCD population hampers further research progress. This manuscript describes the SCDIC (Sickle Cell Disease Implementation Consortium) Registry development and makes the SCDIC Registry baseline and first follow-up data collection forms available for other SCD research efforts.

**Results:**

Study data on 2400 enrolled patients across eight sites was stored and managed using Research Electronic Data Capture (REDCap). Standardized data collection instruments, recruitment and enrollment were refined through consensus of consortium sites. Data points included measures taken from a variety of validated sources (PHENX, PROMIS and others). Surveys were directly administered by research staff and longitudinal follow-up was coordinated through the DCC. Appended registry forms track medical records, event-related patient invalidation, pregnancy, lab reporting, cardiopulmonary and renal functions.

**Conclusions:**

The SCDIC Registry strives to provide an accurate, updated characterization of the adult and adolescent SCD population as well as standardized, validated data collecting tools to guide evidence-based research and practice.

## Background

Sickle cell disease (SCD) is a rare, autosomal recessive blood disorder affecting approximately 100,000 individuals in the United States [1] and 3.1 million individuals worldwide [2]. The scarcity of relevant knowledge and experience with rare diseases creates a unique need for cooperation and infrastructure to overcome challenges in translating basic research advances into clinical advances. Rare disease registries are characterized by systematic, longitudinal collection of clinical, genetic, and biologic data, which can contribute to basic and translational research efforts to improve clinical care for individuals with diseases such as SCD [3].

Over the past decade, SCD registry initiatives in community and clinical settings have employed a variety of methods including electronic-health record abstraction [4], patient-reported outcomes via online portals [5], bio-repositories [6, 7], and collection of clinical data during standard-care visits [8–10]. Some of these registries collect data using validated measures; however, detailed descriptions of the measure selection process and copies of final data collection tools are not available, making it difficult for subsequent researchers to build on previous efforts. These registry initiatives were often limited to a single site or community and were not necessarily representative of the larger SCD population.

The Sickle Cell Disease Implementation Consortium (SCDIC) is a cooperative research program of eight clinical centers, a data coordinating center (DCC), and the National Heart, Lung, and Blood Institute (NHLBI). The SCDIC’s structure and goals are described elsewhere [11]. A key objective of the SCDIC is to develop a registry of at least 2400 adolescents and adults living with SCD. This manuscript describes the SCDIC Registry development and makes the SCDIC Registry baseline and first follow-up data collection forms available for other SCD research efforts.

## Materials/methods

The SCDIC Registry seeks to characterize the SCD population, provide an evidence base to guide research and practice, and leverage existing, validated tools to collect standard clinical measures, laboratory values, lifestyle factors, medical history, treatment, healthcare utilization, and patient reported outcomes. ASCQ-Me [12] and PhenX [13] (SCD-specific measures), and PROMIS [14] measures were used to build the SCDIC Registry forms. Candidate measures were considered and discussed by the SCDIC Registry Subcommittee; selected measures were included in draft data collection forms reviewed by the SCDIC Steering Committee. Standardized data collection forms, and recruitment and enrollment procedures were iteratively refined until consensus was achieved among the consortium sites and NHLBI program staff. Additional file 1 outlines a flow diagram detailing the consortium and study steps towards data collection. Table 1 outlines the SCDIC Registry Timeline. Registry forms and procedures were approved by Institutional Review Boards at each participating institution.

**Table 1:** SCDIC Registry timeline

	Phase I											Phase II				
	Year 1				Year 2				Year 3					Years 4–6		
	Q1	Q2	Q3	Q4	Q1	Q2	Q3	Q4	Q1	Q2		Q3	Q4	Y4	Y5	Y6
**Finalize core measures**		X									**ADMINISTRATIVE REVIEW**					
**Develop procedures**		X	X													
**Design data forms**		X	X													
**Protocol development**			X	X												
**Secure IRB approvals**				X	X											
**Develop data systems**				X	X											
**Manual of operations**				X	X											
**Train coordinators**					X											
**Recruitment and follow-up**					X	X	X	X	X	X		X	X	X	X	X
**Monitoring**						X	X	X	X	X		X	X	X	X	X
**Data analysis and publication**												X	X	X	X	X

### Inclusion criteria

Participants in the SCDIC Registry must be 15 years to 45 years of age and have laboratory confirmed diagnosis of SCD: Hb SS, Hb SC, Hb Sβ-thalassemia, Hb SO, Hb SD, Hb SG, Hb SE, or Hb SF. Participants (or consenting guardian) must have a basic command of English, be willing and cognitively able to give informed consent or assent, and be able to answer questions on the patient enrollment survey.

### Exclusion criteria

Persons unwilling or unable to complete the enrollment survey, persons with sickle cell trait (carriers – Hb AS) and those with a successful bone marrow transplant, are excluded.

## Results

From December 2016 through May 2019, over 2400 adolescents and adults living with SCD were enrolled in the SCDIC Registry across the consortium’s eight clinical sites. All sites reached their goal of 300 enrolled participants and completed baseline data files on each of them. Baseline demographic characteristics, patient reported outcomes, and experiences with SCD-related pain of this cohort are detailed elsewhere [15]. The SCDIC Registry’s baseline and first follow-up data collection procedures and forms are described below. Study data were collected and managed using REDCap (Research Electronic Data Capture) electronic data capture tools hosted at the DCC. REDCap is a secure, web-based application designed to support research study data collection [16]. The SCDIC registry baseline and first follow-up paper forms and REDCap data dictionary are available in the Supplemental Appendices (Additional file 2). Each site employed its own methods to maximize success with longitudinal follow up. Strategies included preset reminders for form completion in REDCap, capturing followup data at routine care appointments, telephone calls to collect survey data, and text message reminders.

### Patient registration form

This form tracks the method of survey administration (interview vs. self-administered), confirmation of the diagnosis of SCD, and basic demographics. Diagnosis status was confirmed by newborn screening, hemoglobin fractionation, hemoglobin electrophoresis or DNA sequencing. Participants are categorized as affiliated or unaffiliated; unaffiliated is defined as not having seen a SCD provider in a non-acute setting in the past 2 years, excluding the enrollment visit.

### Patient enrollment survey form

This form comprises the core of the SCDIC Registry data, covering eight key domains: (1) pain history, (2) hydroxyurea use, (3) blood transfusions, (4) medical history, (5) current medications, (6) barriers to medical care, (7) social and mental health, and (8) detailed demographics. Referent timeframes used measured participants’ chronic and recent SCD-related pain experiences. Questions adapted from published measures maintained face validity, but were not reassessed for construct validity. Table 2 documents the measures reviewed, selected, and adapted. Table 2 documents the measures reviewed, selected, and adapted. In choosing the final measures, the consortium committee sought to avoid redundancy, maximize face and content validity and prioritize measures most likely to be relevant for future research.

**Table 2:** Measures reviewed, selected, and adapted for SCDIC Registry Patient Enrollment Survey Form

	Reviewed	Selected	Adaptations
**ASCQ-Me Pain**	Yes	Yes	
**ASCQ-Me Pain Episode Frequency and Severity**	Yes	Yes	
**ASCQ-Me Stiffness Impact**	Yes	No	
**ASCQ-Me Sleep Impact**	Yes	Partial	Kept items 1 and 3
**ASCQ-ME Sickle Cell Medical History Checklist**	Yes	Yes	
**ASCQ-Me Emotional Impact**	Yes	Partial	Kept items 1 and 5
**ASCQ-Me Social Functioning Impact**	Yes	Yes	
**PhenX Frequency of Sickle Cell Pain Episodes Per Year**	Yes	No	
**PhenX History of Transfusion**	Yes	Yes	
**PROMIS Pain Intensity**	Yes	No	
**PROMIS Fatigue**	Yes	Yes	
**PROMIS Pain Interference**	Yes	No	
**PROMIS Sleep Disturbance**	Yes	No	
**PROMIS-29 Profile (adult)**	Yes	Partial	Kept depression items
**Neuro-QOL Cognitive Function**	Yes	Yes	
**PROMIS PQ-Neuro**	Yes	Partial	Kept item 3
**PROMIS Global Health**	Yes	No	

### Patient follow-up survey form

Longitudinal follow-up is scheduled to occur annually. The follow-up survey is an abbreviated version of the patient enrollment survey and can be administered in person, by phone, by mail, or via an online survey. Three centers are also administering a short questionnaire to obtain the number of adverse events adult participants experienced before turning 18 years old.

### Pregnancy and conception forms

These forms provide information about participants’ conception and pregnancy history, including medical complications, use of hydroxyurea, live births, and fertility treatments (if any) and were designed to capture the impact of hydroxyurea use on reproductive health, especially conception and birth outcomes.

### Medical record abstraction form

Medical record abstraction is completed by research staff for participants with available medical records. Demographics, diagnosis, anthropometric measurements, organ systems review, and treatment are included. A list of definitions used by registry coordinators conducting medical record abstraction is in the Supplemental Appendices.

### Laboratory reporting form

Research staff record the most recent test results at a time the participant was in steady state – defined as at least 2 weeks before or after blood transfusions, vaso-occlusive pain crisis, priapism, stroke, or other acute event.

### Renal form

Research staff complete this form for enrolled participants whose medical abstraction form contains a “Yes” response to Q35 (chronic kidney disease) or Q41 (end stage renal disease). Results are the most recent available within 5 years of the date of consent.

### Pulmonary hypertension and left ventricular dysfunction form

Research staff complete this form for enrolled participants whose medical abstraction form contains a “Yes” response to Q40 (pulmonary hypertension) or Q41 (left ventricular dysfunction). Results are the most recent available within 5 years of the date of consent.

### Off study form

Research staff complete this form for participants who experience an event that invalidates them from follow-up data collection. Cause of death and autopsy reports (if available) are documented for registry participants who died.

## Discussion

Similar studies have characterized SCD populations and provided a wider knowledge base via which the research community could collaborate. The Cooperative Study of Sickle Cell Disease (CSSCD) recruited from 27 sites and provided a frequency of various socioeconomic factors as they relate to mortality and morbidity in this disease population [17]. Unlike the SCDIC and Sickle Cell Clinical Research and Intervention Program (SCCRIP), it had less inclusive criteria, no organ system function analysis or recruitment of patients exposed to disease treatment [18].

SCCRIP like the SCDIC prospectively recruited diagnosed sickle cell disease patients across multiple developmental cohorts (newborn to older adult). It collected clinical, social and health outcomes data for proper characterization of this population with the goal of furthering genomic and proteomic research via identification of treatment-contributing biomarkers [18]. Although similar, the SCDIC has almost twice the sample size, incorporates more validation tools and information systems without requiring a concept proposal for data use, likely making it more publicly accessible. Researchers publishing data using SCDIC forms simply need to acknowledge the Consortium as follows: “Data collection instruments were (used/modified) from those developed under the Sickle Cell Disease Implementation Consortium supported by cooperative agreements from the National Heart, Lung, and Blood Institute and the National Institute on Minority Health and Health Disparities (Bethesda, MD).”

The appended SCDIC Registry forms are of value to SCD research efforts as they (1) are a public resource to facilitate a more modern understanding of this rare disease and its effects on people living with it, (2) are developed from validated measures with broad consensus from SCD and implementation science research experts across the country, (3) form a platform for clinical characterization of people living with SCD in both observational and interventional studies, (4) allow future harmonization of data across studies, and (5) may facilitate mechanistic and therapeutic discoveries in SCD and perhaps other rare diseases. SCDIC registry REDCap data dictionary publication reduces researcher and logistical burden associated with building electronic data capture systems for SCD research.

Data from the SCDIC Registry may serve as a “control” for additional intervention studies, especially when comparison to people living with SCD exposed to hydroxyurea is intended. Additionally, the SCDIC Registry data collection forms serve as a template for new prospective studies in SCD. Information collected on enrolled participants may lead to new potential biomarkers and social determinants for specific outcomes in SCD or allow for the identification of novel study questions. Finally, the SCDIC Registry data will offer an updated description of adolescents and adults with SCD in the United States and may be useful in benchmarking future studies.

## Conclusions

Rare disease registries are characterized by systematic, longitudinal collection of clinical, genetic, and biologic data, which can contribute to basic and translational research efforts to improve clinical care for individuals with diseases such as SCD. The registry forms provided by this study stem from SCD expert-validated measures and may be useful to other researchers conducting interventional or observational studies to further understanding of this disease and its population.

## Supplementary information

**Additional file 1.** Sickle Cell Disease Implementation Consortium flow diagram is provided here.

**Additional file 2:.** “Publication of data collection forms from NHLBI funded Sickle Cell Disease Implementation Consortium (SCDIC) Registry: Supplemental Appendices”. The detailed data collection forms mentioned in the results are provided here.

## Data Availability

The forms supporting this article are included within the article and its additional files.

## Abbreviations

ASCQ-Me: Adult Sickle Cell Quality of Life Measurement (Information System)
DCC: Data Coordinating Center
NHLBI: National Heart, Lung and Blood Institute
PhenX: consensus measures for *Phen*otypes and eXposures
PROMIS: Patient-Reported Outcomes Measurement (Information System)
REDCap: Research Electronic Data Capture
SCD: Sickle Cell Disease
SCDIC: Sickle Cell Disease Implementation Consortium
