# Supplementary figures and images for: Publication of data collection forms from NHLBI funded sickle cell disease implementation consortium (SCDIC) registry
Source: Orphanet J Rare Dis. 2020 Jul 7;15:178. doi: 10.1186/s13023-020-01457-x (PMC7341606; doi:10.1186/s13023-020-01457-x)

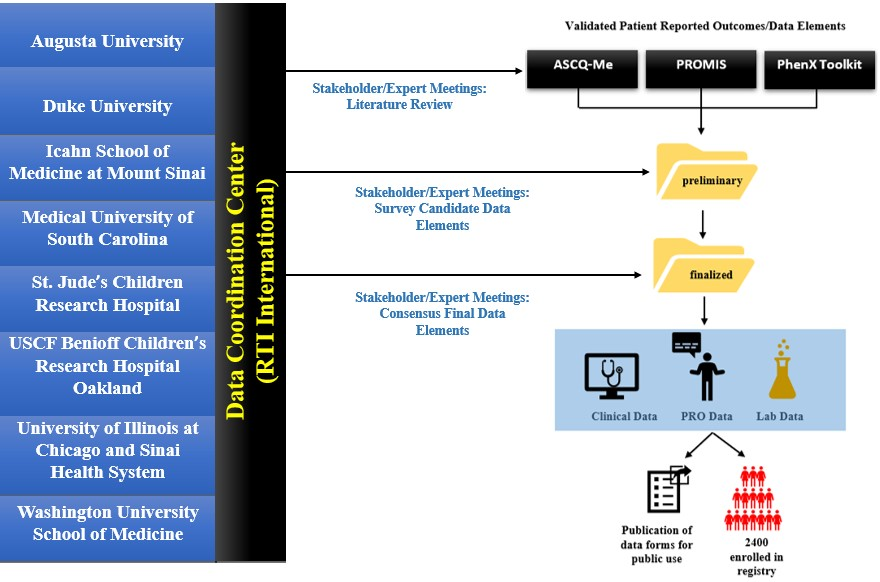

Supplement: Supplementary file 1 — Additional file 1. Sickle Cell Disease Implementation Consortium flow diagram is provided here. [file 13023_2020_1457_MOESM1_ESM.tiff]
