# Supplementary material for: Publication of data collection forms from NHLBI funded sickle cell disease implementation consortium (SCDIC) registry
Source: Orphanet J Rare Dis. 2020 Jul 7;15:178. doi: 10.1186/s13023-020-01457-x (PMC7341606; doi:10.1186/s13023-020-01457-x)
Supplement: Supplementary file 2 — Additional file 2:. “Publication of data collection forms from NHLBI funded Sickle Cell Disease Implementation Consortium (SCDIC) Registry: Supplemental Appendices”. The detailed data collection forms mentioned in the results are provided here. [file 13023_2020_1457_MOESM2_ESM.pdf]

Publication of data collection forms from NHLBI funded Sickle Cell Disease Implementation Consortium (SCDIC) Registry: Supplemental Appendices

Jeffrey A Glassberg [1], Elizabeth A Linton [1], Katrina Burson [2], Tabitha Hendershot [2], Joseph Telfair [3], Julie Kanter [4], Victor R Gordeuk [5], Allison A King [6], Cathy L Melvin [7], Nirmish Shah [8], Jane S Hankins [9], Lynne D Richardson[1], for the Sickle Cell Disease Implementation Consortium

[1]Department of Emergency Medicine, Icahn School of Medicine at Mount Sinai, New York, NY; [2]RTI International, Research Triangle Park, NC; [3]Jiann-Ping Hsu College of Public Health, Georgia Southern University, Statesboro, GA; [4]Division of Hematology & Oncology, University of Alabama at Birmingham School of Medicine, Birmingham, AL; [5]Division of Pediatric Hematology-Oncology, University of Illinois at Chicago, Chicago, IL; [6]Washington University School of Medicine in St. Louis, St. Louis, MO; [7]Department of Public Health Sciences, Medical University of South Carolina, Charleston, SC; [8]Division of Hematology, Duke University School of Medicine, Durham, NC; [9]Department of Hematology, St. Jude Children's Research Hospital, Memphis, TN

Supplemental appendix 1: Definitions of conditions on the Medical Record Abstraction Form 2

Supplemental appendix 2: Members of the Sickle Cell Disease Implementation Consortium 4

Supplemental appendix 3: SCDIC Registry data collection forms (REDCap data dictionary) 5

Supplemental appendix 4: SCDIC Registry data collection forms (Paper) 6

Researchers publishing data collected using SCDIC forms are asked to acknowledge the Consortium as follows: “Data collection instruments were (used/modified) from those developed under the Sickle Cell Disease Implementation Consortium supported by cooperative agreements from the National Heart, Lung, and Blood Institute and the National Institute on Minority Health and Health Disparities (Bethesda, MD)."

Supplemental appendix 1: Definitions of conditions on the Medical Record Abstraction Form

Item	Definition
Steady state	Steady state is 2 weeks before or after blood transfusions, pain crisis, priapism, stroke, or acute event.
**SCD complications section**	
32. Avascular necrosis	1) documented on MRI, or 2) Any mention in medical record is acceptable.
33. Dactylitis	Any mention in medical record is acceptable.
34. Osteomyelitis	Any mention in medical record is acceptable.
35. Chronic kidney disease	1) physician documentation of chronic or abnormal creatinine (>0.8 for Hb SS or Hb SB0>1.2 for Hb SC) on at least two consecutive tests or 2) abnormal creatinine (>0.8 for Hb SS or Hb SB0>1.2 for Hb SC) confirmed at steady state present for at least 3 months (per National Kidney Foundation guidelines).
36. End stage renal disease	1) Having a steady state GFR <15 mL/min/1.73 m2 2) receiving dialysis for at least 3 months, or 3) having undergone kidney transplant.
37. Priapism	Any mention in medical record is acceptable.
38. Stroke	1) documented on MRI, or 2) Any mention in medical record is acceptable.
38d. Silent stroke	Any non-specific bright hypertensive spot on T2 flares requires investigator to adjudicate.
39. Intracranial bleeding	Any mention in medical record is acceptable.
40. Pulmonary arterial hypertension	Any mention of pulmonary hypertension in medical record. An ECHO may be used to confirm the diagnosis but only if it was taken while the person was in steady state.
41. Left ventricular dysfunction	Any mention in medical record is acceptable.
42. Acute chest syndrome	Any mention in medical record is acceptable.
43. Asthma	Any mention in medical record is acceptable.
44. Gallstones/cholelithiasis, cholecystitis	Any mention in medical record is acceptable.
45. Splenomegaly	Any mention in medical record is acceptable.
45c. Hypersplenism	Any mention in medical record is acceptable.
46. Deep vein thrombosis (DVT)	Any mention in medical record is acceptable.
47. Lupus	Any mention in medical record is acceptable
48. Rheumatoid arthritis	Any mention in medical record is acceptable.
49. Gout	Any mention in medical record is acceptable.
50. Sarcoidosis	Any mention in medical record is acceptable.
51. Other autoimmune or Inflammatory	Any mention in medical record is acceptable.
52. Multi-organ failure	Any mention in the medical record is acceptable. Could also be called multi-organ syndrome.
52e. Hemodialysis	'Hemodialysis' may not be stated in the medical record; another acceptable term is Continual Renal Replacement Therapy (CRRT).
53. Pneumococcal sepsis	Any mention in medical record is acceptable.
54. Skin ulcers	Any mention in medical record is acceptable.

Item	Definition
55. Retinopathy	Any mention in medical record is acceptable.
56. Iron overload	Any mention in medical record is acceptable.
57. Chronic refractory pain	1) Received ≥70-day opioid supply in 90-day period, and/or 2) any mention in the medical record is acceptable.
58. Anxiety	Any mention in medical record is acceptable.
59. Depression	Any mention in medical record is acceptable.
60. Other psychiatric disorder	Any mention in medical record is acceptable.
61. Cancer	Any mention in medical record is acceptable.

Supplemental appendix 2: Members of the Sickle Cell Disease Implementation Consortium

ST. JUDE
Jane Hankins, MD, MS
Jason Hodges, PhD, MA
Yvonne Carroll, RN, JD
Lisa Klesges, PhD
Hamda Khan, MA
Matthew Smeltzer, PhD, MS
Chinonyelum Nwosu, MPH
James Gurney, PhD
Jerlym Potter, PhD, MPH
Nicole Alberts, PhD
Jeffrey Frey, BS, MS, MBA, PhD
Michael Jackson
Sherif Badawy, MBBCh, MS
Michael DeBaun, MD, MPH
Ramin Homayouni, PhD
Tamanna Shamrin, PhD
Guolian Kang, PhD
Jeremie Estepp, MD

UCSF
Marsha Treadwell, PhD
Elliott Vichinsky, MD
Ted Wun, MD
Michael Potter, MD
Danielle Hessler, PhD
Ward Hagar, MD
Anne Marsh, MD
Lynne Neumayr, MD

MUSC
Cathy Melvin, PhD
Shannon Phillips, PhD, RN
Robert Adams, MD
Martina Mueller, PhD

DUKE
Nirmish Shah, MD
Paula Tanabe, PhD, MSN
Hayden Bosworth, PhD
George Jackson, PhD
Fred Johnson, MBA
Rachel Richesson, PhD
Janet Prvu-Bettger, ScD

LURIE CHILDRENS
Alexis Thompson, MD

WASH U
Allison King, MD, PhD
Ana Baumann, PhD
Cecelia Calhoun, MD MPHS
Aimee James, PhD, MPH
Regina A. Abel, PhD

AUGUSTA
Abdullah Kutlar, MD
Robert Gibson, PhD
Angie Snyder, PhD
Maria Fernandez, PhD
Matthew Lyon, MD
Richard Lottenberg, MD
Betty Pace, MD
E. Leila Jerome Clay, MD
Raymona Lawrence, DrPH

MOUNT SINAI
Lynne D. Richardson, MD
Jeffrey Glassberg MD, MA
Jena Simon, MS, APRN-BC
Nicholas G. Genes, MD, PhD
George T. Loo, DrPH
Jason S. Shapiro, MD, MA
Kimberly Souffront PhD, FNP-BC, RN
Cindy Clesca, MA
Elizabeth Linton, MPH
Gery Ryan PhD, MA

RTI INTERNATIONAL
Barbara L Kroner, PhD
Lucia Rojas-Smith, DrPH
Tabitha Hendershot, BA
Lisa DiMartino, PhD, MPH
Sara Jacobs, PhD
Whitney Battestilli, BA
Liliana Preiss, MS
Norma Pugh, MS

NHLBI
Sharon M Smith, PhD
Harvey Luksenberg, MD
Marlene Peters-Lawrence, BSN, RN
Cheryl Boyce, PhD
Whitney Barfield, PhD
Ellen Werner, PhD

CHICAGO
Victor Gordeuk, MD
Melissa Gutierrez, MS
Jana Hirschtick, PhD
Lewis Hsu, MD, PhD
Jerry Krishnan, MD, PhD
Nadew Sebro, MD
Abe Wandersman, PhD
Michael Berbaum, PhD
Kishore Bobba, MD
Joe Colla, MD
Kim Erwin, MDes
Andrea Lamont, PhD
Molly Martin, MD, MAPP
Sarah Norell, MDes, MFA
Ananta Pandit, MD
Kay Saving, MD
Robin Shannon, DNP, RN
Robert Winn, MD
Leslie Zun, MD
Judith Nocek, PhD

Supplemental appendix 3: SCDIC Registry data collection forms (REDCap data dictionary)

See attached .csv file.

Supplemental appendix 4: SCDIC Registry data collection forms (Paper)

SCDIC
Sickle Cell Disease Implementation Consortium

Patient Enrollment Survey

Version 1.1 (11/28/2017)

Subject ID

We are interested in learning more about people who have sickle cell disease. As you complete this form, answer the questions as best as you can. If you don't know the answer or do not want to answer a question, you may leave it blank.

1. What is today's date? |__|__|/ |__|__|/ |_2_|_0_|___|___|
   Month Day Year

2. What is your year of birth? |___|___|___|___|
   Year

3. How old are you today? ______ years

4. How old were you when you were diagnosed with sickle cell disease? |___|___| years

5. What type of healthcare professional has been providing the majority of care for your sickle cell disease in the past 2 years?
   - ❒ Sickle cell specialist or hematologist (including all care providers in the SCD clinic)
   - ❒ Primary care or general practice
   - ❒ Emergency department
   - ❒ I don't currently receive care for my sickle cell disease

A. YOUR PAIN HISTORY

6. Do you take pain medicine every day for your sickle cell disease?
   - ❒ Yes
   - ❒ No

7. In the past 12 months, how many sickle cell pain attacks (crises) did you have?
   - ❒ I did not have a pain attack in the past 12 months
   - ❒ 1
   - ❒ 2
   - ❒ 3
   - ❒ 4 or more

8. When was your last pain attack (crisis)?
   - ❒ I've never had a pain attack (crisis)
   - ❒ More than 5 years ago
   - ❒ 1-5 years ago
   - ❒ 7-11 months ago
   - ❒ 1-6 months ago
   - ❒ 1-3 weeks ago
   - ❒ Less than a week ago
   - ❒ I have one right now

9. How severe was your pain during your last pain attack (crisis)? **Circle a number from 0 to 10 below**, where 0 is no pain and 10 is the worst pain imaginable.

0	1	2	3	4	5	6	7	8	9	10

No pain Worst pain imaginable

10. How much did your last pain attack (crisis) interfere with your life?

- ❒ I've never had a pain attack (crisis)
- ❒ Not at all, I did everything I usually do
- ❒ I had to cut down on some things I usually do
- ❒ I could not do most things I usually do
- ❒ I could not take care of myself and needed some help from family or friends
- ❒ I could not take care of myself and needed constant care from family, friends, doctors, or nurses

11. About how long did your most recent pain attack (crisis) last?

- ❒ I've never had a pain attack (crisis)
- ❒ Less than 1 hour
- ❒ 1-12 hours
- ❒ 13-23 hours
- ❒ 1-3 days
- ❒ 4-6 days
- ❒ 1-2 weeks
- ❒ More than 2 weeks

12. Think about your pain in the **past 7 days**, and answer the following questions.

		**Never**	**Rarely**	**Sometimes**	**Often**	**Always**
a.	How often did you have very severe pain?	❒	❒	❒	❒	❒
b.	How often did you have pain so bad that it was hard to finish what you were doing?	❒	❒	❒	❒	❒

13. Now think about your pain in the **past 6 months**, and answer the following questions.

		**Never**	**Rarely**	**Sometimes**	**Often**	**Always**
a.	How often did you have very severe pain?	❒	❒	❒	❒	❒
b.	How often did you have pain so bad that it was hard to finish what you were doing?	❒	❒	❒	❒	❒

14. Think about how your pain felt in the **past 7 days**, and answer the following questions.

		**Not at all**	**A little bit**	**Somewhat**	**Quite a bit**	**Very much**
a.	Did your pain feel like pins and needles?	❒	❒	❒	❒	❒
b.	Did your pain feel sore?	❒	❒	❒	❒	❒

B. YOUR HISTORY OF HYDROXYUREA USE

15. Did a doctor **ever** suggest you take hydroxyurea?

❒ Yes
❒ No

16. What makes it difficult for you to take hydroxyurea or is there a reason why you do not take hydroxyurea? Please select one or more from the list below whether or not you have ever taken hydroxyurea.

❒ I have no difficulties or concerns using hydroxyurea
❒ I don't know enough about the medicine
❒ Sometimes I forget to take the medicine
❒ I am worried about side effects
❒ I don't like the frequent blood tests or clinic visits
❒ I'm feeling well and I don't think I need it
❒ The cost is more than I can afford
❒ I have heard that hydroxyurea may cause cancer
❒ I have heard that hydroxyurea may cause problems with having healthy children
❒ Other difficulty, specify___________________________

17. Have you **ever** taken hydroxyurea?

❒ Yes
❒ No → ***skip to Question 23***

18. Have you experienced any side effects related to hydroxyurea?

❒ Yes
❒ No → ***skip to Question 20***

19. What side effects have you experienced while you were taking hydroxyurea?

❒ Hair loss/thinning
❒ Nail blackening or discoloration
❒ Lowered blood counts (e.g., platelets, white count, hemoglobin)
❒ Low sperm count or other fertility problems
❒ Nausea/vomiting
❒ Skin ulcers
❒ Weight gain
❒ Headaches or dizziness
❒ Fatigue/drowsiness
❒ Other, specify________________________________

20. Are you **currently** on hydroxyurea?

❒ Yes
❒ No → ***skip to Question 22***

21. How many days did you take hydroxyurea in the PAST WEEK?

❒ 0 days
❒ 1 day
❒ 2 days
❒ 3 days
❒ 4 days
❒ 5 days
❒ 6 days
❒ 7 days

Skip to Section C, Question 23 after answering this question

22. What is the reason you discontinued or stopped taking hydroxyurea?

- ❒ Side effects
- ❒ Yours/your family's preference
- ❒ Other reason, specify______________________________

C. YOUR HISTORY OF BLOOD TRANSFUSIONS

23. Do you get regular blood transfusions for your sickle cell disease?

- ❒ Yes
- ❒ No

24. Estimate the number of units (pints) of blood that you have **ever** received.

- ❒ none
- ❒ 1 to 10
- ❒ 11 to 20
- ❒ 21 to 50
- ❒ 50-100
- ❒ more than 100
- ❒ Don't Know

25. Are you on iron chelation treatment **at this time**?

- ❒ Yes
- ❒ No

26. Have you **ever** been told that it is difficult to find blood for you (i.e., you have antibodies or react to other people's blood red blood cells)?

- ❒ Yes
- ❒ No
- ❒ Don't Know

27. Have you **ever** been referred for a bone marrow transplant?

- ❒ Yes
- ❒ No

D. YOUR MEDICAL HISTORY

28. Has a doctor or nurse ever told you that you have or had any of the following conditions? Please check YES or NO for each condition.

	Condition	YES	NO
a.	Lung problems such as pneumonia or acute chest syndrome	❒	❒
b.	Kidney damage	❒	❒
c.	Eye damage called retinopathy	❒	❒
d.	Damage to your hip or shoulder due to sickle cell disease	❒	❒
e.	High blood pressure in your lungs (also called pulmonary hypertension)	❒	❒
f.	Heart failure	❒	❒
g.	Blood clots in your legs or arms or that went to your lung	❒	❒
h.	A stroke	❒	❒
i.	Asthma	❒	❒
j.	Diabetes	❒	❒

29. Have you ever had open sores on your legs or feet (leg ulcers)?

- ❒ Yes
- ❒ No

30. Has your spleen either been removed or seriously damaged due to sickle cell disease?

- ❒ Yes
- ❒ No

E. MEDICATIONS YOU ARE TAKING AT THE PRESENT TIME

31. Please list all medications you are **currently** taking.

Name of Medication	Name of Medication
1.	6.
2.	7.
3.	8.
4.	9.
5.	10.

F. BARRIERS TO YOUR MEDICAL CARE

32. During the past 12 months, was there any time when you didn't get the medical care you needed or had delays in getting the care you needed?

- ❒ Yes
- ❒ No → ***skip to Question 34***

33. Did you not get the medical care you needed or have delays getting medical care you needed for any of the following reasons?

- ❒ Worry about the cost
- ❒ The doctor or hospital wouldn‘t accept your health insurance
- ❒ Your health plan wouldn‘t pay for the treatment
- ❒ You couldn‘t get an appointment soon enough
- ❒ You couldn‘t get there when the doctor‘s office or clinic was open
- ❒ It takes too long to get to the doctor‘s office or clinic from your house or work
- ❒ You couldn‘t get through on the telephone
- ❒ You were too busy with work or other commitments to take the time
- ❒ You didn‘t think the problem was serious enough
- ❒ You had previous bad experiences with the health care system
- ❒ People at the doctor’s office or clinic don’t speak the same language I do
- ❒ Some other reason not listed above, please specify _______________________________

G. YOUR SOCIAL AND MENTAL HEALTH

34. Think about your sleep in the **past 7 days**, and answer the following questions.

		Never	Rarely	Sometimes	Often	Always
a.	How often did you stay up most of the night because you could not fall asleep?	❒	❒	❒	❒	❒
b.	How often did you have a lot of trouble falling asleep?	❒	❒	❒	❒	❒

35. In the **past 7 days**, how often did the following happen?

		Never	Rarely (Once)	Sometimes (2-3 times)	Often (once a day)	Very often (several times a day)
a.	I had to read something several times to understand it.	❒	❒	❒	❒	❒
b.	My thinking was slow.	❒	❒	❒	❒	❒
c.	I had to work really hard to pay attention or I would make a mistake.	❒	❒	❒	❒	❒
d.	I had trouble concentrating.	❒	❒	❒	❒	❒

36. How much DIFFICULTY do you **currently** have doing the following things?

		None	A little	Somewhat	A lot	Cannot do
a.	Reading and following complex instructions (e.g., directions for a new medication)?	❒	❒	❒	❒	❒
b.	Planning for and keeping appointments that are not part of your weekly routine (e.g, a therapy or doctor appointment, a social gathering with friends or family)?	❒	❒	❒	❒	❒
c.	Managing your time to do most of your daily activities?	❒	❒	❒	❒	❒
d.	Learning new tasks or instructions?	❒	❒	❒	❒	❒

37. Think about how you felt in the **past 7 days**, and respond to each question or statement.

		Never	Rarely	Sometimes	Often	Always
a.	I felt worthless.	❒	❒	❒	❒	❒
b.	I felt helpless.	❒	❒	❒	❒	❒
c.	I felt depressed.	❒	❒	❒	❒	❒
d.	I felt hopeless.	❒	❒	❒	❒	❒
e.	How often did you feel completely hopeless because of your health?	❒	❒	❒	❒	❒
f.	How often were you very worried about needing to go to the hospital?	❒	❒	❒	❒	❒
		**Not at all**	**A little bit**	**Somewhat**	**Quite a bit**	**Very much**
g.	I felt tired.	❒	❒	❒	❒	❒

38. Have you ever been treated for depression?

- ❒ Yes, currently receiving treatment
- ❒ Yes, treated in the past but not now
- ❒ No, never received treatment

39. In the **past 30 days**, how much did the following happen?

		Not at all	A little bit	Somewhat	Quite a bit	Very much
a.	How much did you rely on others to take care of you because of your health?	❒	❒	❒	❒	❒
b.	How much did your health make it hard for you to do things with your friends?	❒	❒	❒	❒	❒

H. TELL US ABOUT YOURSELF

40. Are you male or female?

❒ Male
❒ Female

41. Do you consider yourself Hispanic/Latino or not Hispanic/Latino?

❒ Hispanic or Latino
❒ Not Hispanic or Latino

42. Which of the following five racial designations best describes you? More than one choice is acceptable.

❒ American Indian or Alaska Native
❒ Asian
❒ Black or African American
❒ Native Hawaiian or Pacific Islander
❒ White

43. In what language do you feel most comfortable speaking with your doctor or nurse?

❒ English
❒ Spanish
❒ Another language

44. What is your current marital status?

❒ Not Applicable (subject is a child)
❒ Married
❒ Living as married (including living with a partner)
❒ Divorced or separated
❒ Widowed
❒ Never married

45. How many children and adults, including yourself, live in your household at least 4 nights a week?

_______ # of children _______ # of adults

46. What is your approximate yearly household income? Include income from all sources.

❒ $25,000 and under
❒ $25,001 - $50,000
❒ $50,001 - $75,000
❒ $75,001 - $100,000
❒ >$100,000

47. What is the highest grade or level of school you have completed or the highest degree you have received?

- ❒ Less than High School
- ❒ Some high school
- ❒ High school graduate or GED equivalent
- ❒ Some college or vocational training
- ❒ College graduate
- ❒ Some graduate school or professional school
- ❒ Graduate or professional degree

48. We would like to know about what you do -- are you working, looking for work, retired, keeping house, or what?

- ❒ Working now
- ❒ Only temporarily laid off, sick leave, or maternity leave
- ❒ Looking for work, unemployed
- ❒ Retired
- ❒ Disabled, permanently or temporarily
- ❒ Keeping house
- ❒ Student
- ❒ Other (Specify):______________________

This is the END of the survey. Please return it to the study coordinator.
Thank you for your participation.

SUBJECT ID

PREGNANCY AND CONCEPTION FORM

For Females

Final Version 1.1, 11/28/2017

This form asks questions about pregnancies you have had.

1. Have you ever been pregnant?

   ☐ No → **SKIP TO QUESTION 13 ON THE BACK OF THIS FORM**
   ☐ Yes

2. How many times have you been pregnant? Please be sure to include any pregnancies that ended in a live birth, miscarriage, stillbirth, or abortion. Enter the total number on the line below.

   _____ total number of pregnancies in your lifetime

INSTRUCTIONS FOR PAGES 2-3:

As you answer the questions on the following 2 pages, please think about each of the pregnancies that you have had. Start with the earliest pregnancy, listing it in the first column labeled "1st pregnancy". From there, work forward until you have provided information about all of the pregnancies you listed in question 2 above. Then go to the back page and answer the remaining questions. Tell the study coordinator if you have had more than 6 pregnancies.

		1st pregnancy	2nd pregnancy	3rd pregnancy
**3.**	In what month and year did this pregnancy <u>end</u> (enter due date if currently pregnant)?	______ / _________<br>Month / Year	______ / _________<br>Month / Year	______ / _________<br>Month / Year
**4.**	What was the outcome of this pregnancy?	☐ Live birth<br>☐ Still birth<br>☐ Miscarriage<br>☐ Abortion<br>☐ Currently pregnant	☐ Live birth<br>☐ Still birth<br>☐ Miscarriage<br>☐ Abortion<br>☐ Currently pregnant	☐ Live birth<br>☐ Still birth<br>☐ Miscarriage<br>☐ Abortion<br>☐ Currently pregnant
**5.**	Were you taking hydroxyurea at the <u>time of conception</u> (when the pregnancy started) or within the month before conception?	☐ No<br>☐ Yes<br>☐ Don't remember	☐ No<br>☐ Yes<br>☐ Don't remember	☐ No<br>☐ Yes<br>☐ Don't remember
**6.**	<u>During this pregnancy</u> were you taking hydroxyurea?<br>*If yes, check all trimesters that apply or that you can remember.*	☐ No, did not take HU<br>☐ Yes, during 1st trimester<br>☐ Yes, during 2st trimester<br>☐ Yes, during 3rd trimester<br>☐ Don't remember	☐ No, did not take HU<br>☐ Yes, during 1st trimester<br>☐ Yes, during 2st trimester<br>☐ Yes, during 3rd trimester<br>☐ Don't remember	☐ No, did not take HU<br>☐ Yes, during 1st trimester<br>☐ Yes, during 2st trimester<br>☐ Yes, during 3rd trimester<br>☐ Don't remember
**7.**	Did you take any fertility drugs or receive any procedure from a health care worker to help you get pregnant with this pregnancy?	☐ No<br>☐ Yes	☐ No<br>☐ Yes	☐ No<br>☐ Yes

**** Answer Questions 8 – 12 below only if the pregnancy ended in a live birth**

		1st pregnancy	2nd pregnancy	3rd pregnancy
**8.**	How many babies were born with this pregnancy?	______ # of babies	______ # of babies	______ # of babies
**9.**	Was the baby (or babies) born prematurely?	☐ No, not born prematurely<br>☐ Yes → enter how many weeks of gestation ________	☐ No, not born prematurely<br>☐ Yes → enter how many weeks of gestation ________	☐ No, not born prematurely<br>☐ Yes → enter how many weeks of gestation ________
**10.**	Did any of the babies in this pregnancy weigh **less than 5.5 pounds** at the time of birth?	☐ No<br>☐ Yes	☐ No<br>☐ Yes	☐ No<br>☐ Yes
**11.**	Did a doctor ever say a baby from this pregnancy had low birth weight, a birth defect, a genetic condition, or another serious medical problem related to birth?	☐ No<br>☐ Yes → What condition?	☐ No<br>☐ Yes→ What condition?	☐ No<br>☐ Yes→ What condition?
**12.**	Did you have any significant medical complications during this pregnancy?<br>*Check all that apply*	☐ No complications<br>☐ Pain crisis<br>☐ Acute chest syndrome<br>☐ Preeclampsia<br>☐ Maternal diabetes<br>☐ Transfusion required<br>☐ Blood clots<br>☐ Other specify __________	☐ No complications<br>☐ Pain crisis<br>☐ Acute chest syndrome<br>☐ Preeclampsia<br>☐ Maternal diabetes<br>☐ Transfusion required<br>☐ Blood clots<br>☐ Other specify __________	☐ No complications<br>☐ Pain crisis<br>☐ Acute chest syndrome<br>☐ Preeclampsia<br>☐ Maternal diabetes<br>☐ Transfusion required<br>☐ Blood clots<br>☐ Other specify __________

		4th pregnancy	5th pregnancy	6th pregnancy
3.	In what month and year did this pregnancy end (enter due date if currently pregnant)?	______ / _________<br>Month / Year	______ / _________<br>Month / Year	______ / _________<br>Month / Year
4.	What was the outcome of this pregnancy?	☐ Live birth<br>☐ Still birth<br>☐ Miscarriage<br>☐ Abortion<br>☐ Currently pregnant	☐ Live birth<br>☐ Still birth<br>☐ Miscarriage<br>☐ Abortion<br>☐ Currently pregnant	☐ Live birth<br>☐ Still birth<br>☐ Miscarriage<br>☐ Abortion<br>☐ Currently pregnant
5.	Were you taking hydroxyurea at the time of conception (when the pregnancy started) or within the month before conception?	☐ No<br>☐ Yes<br>☐ Don't remember	☐ No<br>☐ Yes<br>☐ Don't remember	☐ No<br>☐ Yes<br>☐ Don't remember
6.	During this pregnancy were you taking hydroxyurea?<br>*If yes, check all trimesters that apply or that you can remember.*	☐ No, did not take HU<br>☐ Yes, during 1st trimester<br>☐ Yes, during 2st trimester<br>☐ Yes, during 3rd trimester<br>☐ Don't remember	☐ No, did not take HU<br>☐ Yes, during 1st trimester<br>☐ Yes, during 2st trimester<br>☐ Yes, during 3rd trimester<br>☐ Don't remember	☐ No, did not take HU<br>☐ Yes, during 1st trimester<br>☐ Yes, during 2st trimester<br>☐ Yes, during 3rd trimester<br>☐ Don't remember
7.	Did you take any fertility drugs or receive any procedure from a health care worker to help you get pregnant with this pregnancy?	☐ No<br>☐ Yes	☐ No<br>☐ Yes	☐ No<br>☐ Yes
**** Answer Questions 8 – 12 below only if the pregnancy ended in a live birth**				
		**4th pregnancy**	**5th pregnancy**	**6th pregnancy**
8.	How many babies were born with this pregnancy?	______ # of babies	______ # of babies	______ # of babies
9.	Was the baby (or babies) born prematurely?	☐ No, not born prematurely<br>☐ Yes → enter how many weeks of gestation ________	☐ No, not born prematurely<br>☐ Yes → enter how many weeks of gestation ________	☐ No, not born prematurely<br>☐ Yes → enter how many weeks of gestation ________
10.	Did any of the babies in this pregnancy weigh **less than 5.5 pounds** at the time of birth?	☐ No<br>☐ Yes	☐ No<br>☐ Yes	☐ No<br>☐ Yes
11.	Did a doctor ever say a baby from this pregnancy had low birth weight, a birth defect, a genetic condition, or another serious medical problem related to birth?	☐ No<br>☐ Yes → What condition?	☐ No<br>☐ Yes→ What condition?	☐ No<br>☐ Yes→ What condition?
12.	Did you have any significant medical complications during this pregnancy?<br>*Check all that apply*	☐ No complications<br>☐ Pain crisis<br>☐ Acute chest syndrome<br>☐ Preeclampsia<br>☐ Maternal diabetes<br>☐ Transfusion required<br>☐ Blood clots<br>☐ Other specify __________	☐ No complications<br>☐ Pain crisis<br>☐ Acute chest syndrome<br>☐ Preeclampsia<br>☐ Maternal diabetes<br>☐ Transfusion required<br>☐ Blood clots<br>☐ Other specify __________	☐ No complications<br>☐ Pain crisis<br>☐ Acute chest syndrome<br>☐ Preeclampsia<br>☐ Maternal diabetes<br>☐ Transfusion required<br>☐ Blood clots<br>☐ Other specify __________

13. Has there ever been a time in your life during which you didn't become pregnant despite 12 or more months of regular unprotected intercourse?

- ☐ No → **SKIP TO END**
- ☐ Yes

14. Did you ever go to a doctor or other medical care provider to talk about ways to help you have a baby?

- ☐ Yes
- ☐ No → **GO TO QUESTION 16**

15. Which of the services did you have to help you have a baby? Check all the apply.

- ☐ Advice
- ☐ Infertility testing
- ☐ Drugs to improve ovulation
- ☐ Surgery to correct blocked tubes
- ☐ Artificial insemination
- ☐ Other types of medical help

16. Has a doctor or other medical care provider ever told you that you had fibroid tumors or myomas in your uterus?

- ☐ Yes
- ☐ No

17. Has a doctor or other medical care provider ever told you that you had endometriosis?

- ☐ Yes
- ☐ No

THIS IS THE END OF THE FORM. THANK YOU FOR YOUR PARTICIPATION.

PLEASE RETURN THE FORM TO THE STUDY COORDINATOR.

Instructions to Coordinator: Use this form for subjects with more than 6 pregnancies. Record the number of additional pregnancies and give this form to the patient to provide the information on the additional pregnancies.

		**____ pregnancy**	**____ pregnancy**	**____ pregnancy**
**3.**	In what month and year did this pregnancy end (enter due date if currently pregnant)?	______ / _________<br>Month / Year	______ / _________<br>Month / Year	______ / _________<br>Month / Year
**4.**	What was the outcome of this pregnancy?	☐ Live birth<br>☐ Still birth<br>☐ Miscarriage<br>☐ Abortion<br>☐ Currently pregnant	☐ Live birth<br>☐ Still birth<br>☐ Miscarriage<br>☐ Abortion<br>☐ Currently pregnant	☐ Live birth<br>☐ Still birth<br>☐ Miscarriage<br>☐ Abortion<br>☐ Currently pregnant
**5.**	Were you taking hydroxyurea at the time of conception (when the pregnancy started) or within the month before conception?	☐ No<br>☐ Yes<br>☐ Don't remember	☐ No<br>☐ Yes<br>☐ Don't remember	☐ No<br>☐ Yes<br>☐ Don't remember
**6.**	During this pregnancy were you taking hydroxyurea?<br>*If yes, check all trimesters that apply or that you can remember.*	☐ No, did not take HU<br>☐ Yes, during 1st trimester<br>☐ Yes, during 2st trimester<br>☐ Yes, during 3rd trimester<br>☐ Don't remember	☐ No, did not take HU<br>☐ Yes, during 1st trimester<br>☐ Yes, during 2st trimester<br>☐ Yes, during 3rd trimester<br>☐ Don't remember	☐ No, did not take HU<br>☐ Yes, during 1st trimester<br>☐ Yes, during 2st trimester<br>☐ Yes, during 3rd trimester<br>☐ Don't remember
**7.**	Did you take any fertility drugs or receive any procedure from a health care worker to help you get pregnant with this pregnancy?	☐ No<br>☐ Yes	☐ No<br>☐ Yes	☐ No<br>☐ Yes

**** Answer Questions 8 – 12 below only if the pregnancy ended in a live birth**

		**____ pregnancy (same # as above)**	**____ pregnancy (same # as above)**	**____ pregnancy (same # as above)**
**8.**	How many babies were born with this pregnancy?	______ # of babies	______ # of babies	______ # of babies
**9.**	Was the baby (or babies) born prematurely?	☐ No, not born prematurely<br>☐ Yes → enter how many weeks of gestation ________	☐ No, not born prematurely<br>☐ Yes → enter how many weeks of gestation ________	☐ No, not born prematurely<br>☐ Yes → enter how many weeks of gestation ________
**10.**	Did any of the babies in this pregnancy weigh **less than 5.5 pounds** at the time of birth?	☐ No<br>☐ Yes	☐ No<br>☐ Yes	☐ No<br>☐ Yes
**11.**	Did a doctor ever say a baby from this pregnancy had low birth weight, a birth defect, a genetic condition, or another serious medical problem related to birth?	☐ No<br>☐ Yes → What condition?	☐ No<br>☐ Yes→ What condition?	☐ No<br>☐ Yes→ What condition?
**12.**	Did you have any significant medical complications during this pregnancy?<br>*Check all that apply*	☐ No complications<br>☐ Pain crisis<br>☐ Acute chest syndrome<br>☐ Preeclampsia<br>☐ Maternal diabetes<br>☐ Transfusion required<br>☐ Blood clots<br>☐ Other specify __________	☐ No complications<br>☐ Pain crisis<br>☐ Acute chest syndrome<br>☐ Preeclampsia<br>☐ Maternal diabetes<br>☐ Transfusion required<br>☐ Blood clots<br>☐ Other specify __________	☐ No complications<br>☐ Pain crisis<br>☐ Acute chest syndrome<br>☐ Preeclampsia<br>☐ Maternal diabetes<br>☐ Transfusion required<br>☐ Blood clots<br>☐ Other specify __________

		____ pregnancy	____ pregnancy	____ pregnancy
3.	In what month and year did this pregnancy end (enter due date if currently pregnant)?	______ / _________<br>Month / Year	______ / _________<br>Month / Year	______ / _________<br>Month / Year
4.	What was the outcome of this pregnancy?	☐ Live birth<br>☐ Still birth<br>☐ Miscarriage<br>☐ Abortion<br>☐ Currently pregnant	☐ Live birth<br>☐ Still birth<br>☐ Miscarriage<br>☐ Abortion<br>☐ Currently pregnant	☐ Live birth<br>☐ Still birth<br>☐ Miscarriage<br>☐ Abortion<br>☐ Currently pregnant
5.	Were you taking hydroxyurea at the time of conception (when the pregnancy started) or within the month before conception?	☐ No<br>☐ Yes<br>☐ Don't remember	☐ No<br>☐ Yes<br>☐ Don't remember	☐ No<br>☐ Yes<br>☐ Don't remember
6.	During this pregnancy were you taking hydroxyurea?<br>*If yes, check all trimesters that apply or that you can remember.*	☐ No, did not take HU<br>☐ Yes, during 1st trimester<br>☐ Yes, during 2st trimester<br>☐ Yes, during 3rd trimester<br>☐ Don't remember	☐ No, did not take HU<br>☐ Yes, during 1st trimester<br>☐ Yes, during 2st trimester<br>☐ Yes, during 3rd trimester<br>☐ Don't remember	☐ No, did not take HU<br>☐ Yes, during 1st trimester<br>☐ Yes, during 2st trimester<br>☐ Yes, during 3rd trimester<br>☐ Don't remember
7.	Did you take any fertility drugs or receive any procedure from a health care worker to help you get pregnant with this pregnancy?	☐ No<br>☐ Yes	☐ No<br>☐ Yes	☐ No<br>☐ Yes

**** Answer Questions 8 – 12 below only if the pregnancy ended in a live birth**

		____ pregnancy (same # as above)	____ pregnancy (same # as above)	____ pregnancy (same # as above)
8.	How many babies were born with this pregnancy?	______ # of babies	______ # of babies	______ # of babies
9.	Was the baby (or babies) born prematurely?	☐ No, not born prematurely<br>☐ Yes → enter how many weeks of gestation ________	☐ No, not born prematurely<br>☐ Yes → enter how many weeks of gestation ________	☐ No, not born prematurely<br>☐ Yes → enter how many weeks of gestation ________
10.	Did any of the babies in this pregnancy weigh **less than 5.5 pounds** at the time of birth?	☐ No<br>☐ Yes	☐ No<br>☐ Yes	☐ No<br>☐ Yes
11.	Did a doctor ever say a baby from this pregnancy had low birth weight, a birth defect, a genetic condition, or another serious medical problem related to birth?	☐ No<br>☐ Yes → What condition?	☐ No<br>☐ Yes→ What condition?	☐ No<br>☐ Yes→ What condition?
12.	Did you have any significant medical complications during this pregnancy?<br>*Check all that apply*	☐ No complications<br>☐ Pain crisis<br>☐ Acute chest syndrome<br>☐ Preeclampsia<br>☐ Maternal diabetes<br>☐ Transfusion required<br>☐ Blood clots<br>☐ Other specify __________	☐ No complications<br>☐ Pain crisis<br>☐ Acute chest syndrome<br>☐ Preeclampsia<br>☐ Maternal diabetes<br>☐ Transfusion required<br>☐ Blood clots<br>☐ Other specify __________	☐ No complications<br>☐ Pain crisis<br>☐ Acute chest syndrome<br>☐ Preeclampsia<br>☐ Maternal diabetes<br>☐ Transfusion required<br>☐ Blood clots<br>☐ Other specify __________

PREGNANCY AND CONCEPTION FORM
For Males

Final Version 1.1, 11/286/2017

Subject ID

This form asks questions about pregnancies where you have been the father.

1. Have you ever fathered a baby?

   ☐ No → **SKIP TO QUESTION 10 ON THE BACK OF THIS FORM**
   ☐ Yes

2. How many times have you fathered a baby? Please be sure to include any pregnancies that are current or ended in a live birth, miscarriage, stillbirth, or abortion. Enter the total number on the line below.

   _____ total number of pregnancies where you have been the father

INSTRUCTIONS FOR QUESTIONS 3-9:
As you answer the questions on the following 2 pages, please think about each of the pregnancies where you have been the father. Start with the earliest pregnancy, listing it in the first column labeled "1st pregnancy". From there, work forward until you have provided information about all of the pregnancies you listed in question 2 above. Then go to the back page and answer the remaining questions. Tell the study coordinator if you have fathered more than 8 pregnancies.

		1st pregnancy	2nd pregnancy	3rd pregnancy
**3.**	In what month and year did this pregnancy end (or due date if currently pregnancy)?	______ / _________<br>Month / Year	______ / _________<br>Month / Year	______ / _________<br>Month / Year
**4.**	What was the outcome of this pregnancy?	☐ Live birth<br>☐ Still birth<br>☐ Miscarriage<br>☐ Abortion<br>☐ Currently pregnant	☐ Live birth<br>☐ Still birth<br>☐ Miscarriage<br>☐ Abortion<br>☐ Currently pregnant	☐ Live birth<br>☐ Still birth<br>☐ Miscarriage<br>☐ Abortion<br>☐ Currently pregnant
**5.**	Were you taking hydroxyurea at the time of conception (when the pregnancy started) or within the month before conception?	☐ No<br>☐ Yes<br>☐ Don't remember	☐ No<br>☐ Yes<br>☐ Don't remember	☐ No<br>☐ Yes<br>☐ Don't remember
**** Answer Questions 6 – 9 below only if the pregnancy ended in a live birth**				
		**1st pregnancy**	**2nd pregnancy**	**3rd pregnancy**
**6.**	How many babies were born with this pregnancy?	______ # of babies	______ # of babies	______ # of babies
**7.**	Was the baby (or babies) born prematurely?	☐ No, not born prematurely<br>☐ Yes → enter how many weeks of gestation ________	☐ No, not born prematurely<br>☐ Yes → enter how many weeks of gestation ________	☐ No, not born prematurely<br>☐ Yes → enter how many weeks of gestation ________
**8.**	Did any of the babies in this pregnancy weigh **less than 5.5 pounds** at the time of birth?	☐ No<br>☐ Yes<br>☐ Don't know	☐ No<br>☐ Yes<br>☐ Don't know	☐ No<br>☐ Yes<br>☐ Don't know
**9.**	Did a doctor ever say a baby from this pregnancy had low birth weight, a birth defect, a genetic condition, or another serious medical problem related to birth?	☐ No<br>☐ Yes → What condition?	☐ No<br>☐ Yes→ What condition?	☐ No<br>☐ Yes→ What condition?

		4th pregnancy	5th pregnancy	6th pregnancy
**3.**	In what month and year did this pregnancy end (or due date if currently pregnant)?	______ / _________<br>Month / Year	______ / _________<br>Month / Year	______ / _________<br>Month / Year
**4.**	What was the outcome of this pregnancy?	☐ Live birth<br>☐ Still birth<br>☐ Miscarriage<br>☐ Abortion<br>☐ Currently pregnant	☐ Live birth<br>☐ Still birth<br>☐ Miscarriage<br>☐ Abortion<br>☐ Currently pregnant	☐ Live birth<br>☐ Still birth<br>☐ Miscarriage<br>☐ Abortion<br>☐ Currently pregnant
**5.**	Were you taking hydroxyurea at the time of conception (when the pregnancy started) or within the month before conception?	☐ No<br>☐ Yes<br>☐ Don't remember	☐ No<br>☐ Yes<br>☐ Don't remember	☐ No<br>☐ Yes<br>☐ Don't remember

**** Answer Questions 6 – 9 below only if the pregnancy ended in a live birth**

		4th pregnancy	5th pregnancy	6th pregnancy
**6.**	How many babies were born with this pregnancy?	______ # of babies	______ # of babies	______ # of babies
**7.**	Was the baby (or babies) born prematurely?	☐ No, not born prematurely<br>☐ Yes → enter how many weeks of gestation ________	☐ No, not born prematurely<br>☐ Yes → enter how many weeks of gestation ________	☐ No, not born prematurely<br>☐ Yes → enter how many weeks of gestation ________
**8.**	Did any of the babies in this pregnancy weigh **less than 5.5 pounds** at the time of birth?	☐ No<br>☐ Yes<br>☐ Don't know	☐ No<br>☐ Yes<br>☐ Don't know	☐ No<br>☐ Yes<br>☐ Don't know
**9.**	Did a doctor ever say a baby from this pregnancy had low birth weight, a birth defect, a genetic condition, or another serious medical problem related to birth?	☐ No<br>☐ Yes → What condition?	☐ No<br>☐ Yes→ What condition?	☐ No<br>☐ Yes→ What condition?

10. Have you ever had a painful continuous erection, which is also called priapism?

    ☐ No
    ☐ Yes

11. Has there ever been a time in your life during which you weren't able to get your partner pregnant despite 12 or more months of regular unprotected intercourse?

    ☐ No → **FORM COMPLETE**
    ☐ Yes

12. Did you ever go to a doctor or other medical care provider to talk about ways to help you father a baby?

    ☐ No → **FORM COMPLETE**
    ☐ Yes → **GO TO QUESTION 13**

13. Which of the following services did you have to help you father a baby? Check all the apply.

    ☐ Advice
    ☐ Infertility testing
    ☐ Surgery to reverse a vasectomy
    ☐ Treatment for varicocele
    ☐ Other types of medical help

14. When you went for medical help to father a baby, were you ever told that you had any of the following male infertility problems? Check all that apply.

    ☐ Sperm or semen problems
    ☐ Varicocele
    ☐ Other
    ☐ None of the above

This is the END of the survey. Thank you for your participation. Please return the form to the study coordinator.

Instructions to Coordinator: Use this form for subjects with more than 6 pregnancies. Record the number of additional pregnancies and give this form to the patient to provide the information on the additional pregnancies .

		**____ pregnancy**	**____ pregnancy**	**____ pregnancy**
**3.**	In what month and year did this pregnancy end (or due date if currently pregnancy)?	______ / _________<br>Month / Year	______ / _________<br>Month / Year	______ / _________<br>Month / Year
**4.**	What was the outcome of this pregnancy?	☐ Live birth<br>☐ Still birth<br>☐ Miscarriage<br>☐ Abortion<br>☐ Currently pregnant	☐ Live birth<br>☐ Still birth<br>☐ Miscarriage<br>☐ Abortion<br>☐ Currently pregnant	☐ Live birth<br>☐ Still birth<br>☐ Miscarriage<br>☐ Abortion<br>☐ Currently pregnant
**5.**	Were you taking hydroxyurea at the time of conception (when the pregnancy started) or within the month before conception?	☐ No<br>☐ Yes<br>☐ Don't remember	☐ No<br>☐ Yes<br>☐ Don't remember	☐ No<br>☐ Yes<br>☐ Don't remember
**** Answer Questions 6 – 9 below only if the pregnancy ended in a live birth**				
		**____ pregnancy (same # as above)**	**____ pregnancy (same # as above)**	**____ pregnancy (same # as above)**
**6.**	How many babies were born with this pregnancy?	______ # of babies	______ # of babies	______ # of babies
**7.**	Was the baby (or babies) born prematurely?	☐ No, not born prematurely<br>☐ Yes → enter how many weeks of gestation ________	☐ No, not born prematurely<br>☐ Yes → enter how many weeks of gestation ________	☐ No, not born prematurely<br>☐ Yes → enter how many weeks of gestation ________
**8.**	Did any of the babies in this pregnancy weigh **less than 5.5 pounds** at the time of birth?	☐ No<br>☐ Yes<br>☐ Don't know	☐ No<br>☐ Yes<br>☐ Don't know	☐ No<br>☐ Yes<br>☐ Don't know
**9.**	Did a doctor ever say a baby from this pregnancy had low birth weight, a birth defect, a genetic condition, or another serious medical problem related to birth?	☐ No<br>☐ Yes → What condition?	☐ No<br>☐ Yes→ What condition?	☐ No<br>☐ Yes→ What condition?

		____ pregnancy	____ pregnancy	____ pregnancy
**3.**	In what month and year did this pregnancy end (or due date if currently pregnant)?	______ / _________<br>Month / Year	______ / _________<br>Month / Year	______ / _________<br>Month / Year
**4.**	What was the outcome of this pregnancy?	☐ Live birth<br>☐ Still birth<br>☐ Miscarriage<br>☐ Abortion<br>☐ Currently pregnant	☐ Live birth<br>☐ Still birth<br>☐ Miscarriage<br>☐ Abortion<br>☐ Currently pregnant	☐ Live birth<br>☐ Still birth<br>☐ Miscarriage<br>☐ Abortion<br>☐ Currently pregnant
**5.**	Were you taking hydroxyurea at the time of conception (when the pregnancy started) or within the month before conception?	☐ No<br>☐ Yes<br>☐ Don't remember	☐ No<br>☐ Yes<br>☐ Don't remember	☐ No<br>☐ Yes<br>☐ Don't remember
**** Answer Questions 6 – 9 below only if the pregnancy ended in a live birth**				
		**____ pregnancy (same # as above)**	**____ pregnancy (same # as above)**	**____ pregnancy (same # as above)**
**6.**	How many babies were born with this pregnancy?	______ # of babies	______ # of babies	______ # of babies
**7.**	Was the baby (or babies) born prematurely?	☐ No, not born prematurely<br>☐ Yes → enter how many weeks of gestation ________	☐ No, not born prematurely<br>☐ Yes → enter how many weeks of gestation ________	☐ No, not born prematurely<br>☐ Yes → enter how many weeks of gestation ________
**8.**	Did any of the babies in this pregnancy weigh **less than 5.5 pounds** at the time of birth?	☐ No<br>☐ Yes<br>☐ Don't know	☐ No<br>☐ Yes<br>☐ Don't know	☐ No<br>☐ Yes<br>☐ Don't know
**9.**	Did a doctor ever say a baby from this pregnancy had low birth weight, a birth defect, a genetic condition, or another serious medical problem related to birth?	☐ No<br>☐ Yes → What condition?	☐ No<br>☐ Yes→ What condition?	☐ No<br>☐ Yes→ What condition?

Subject ID Label

Patient Registration Form

Version 2 (7/2/2018)

By entering this form into the DMS, you are entering this subject into the SCDIC Registry database. The REDCap survey is accessible after the SCD diagnosis status is entered. Demographics should be completed for eligible subjects only.

Registration Checklist:

- ☐ The subject provided signed consent to participate in the Registry on ______________.
  (DATE)
  - ☐ Assent form signed (minors only)
- ☐ The subject completed the patient survey via the following mode (check one):
  - ☐ Interview; hard copy
  - ☐ Interview; online entry
  - ☐ Interview: phone
  - ☐ Self-administered; hard copy
  - ☐ Self-administered; online entry

<u>Diagnosis Status:</u>

- ☐ Confirmed (with documentation) by newborn screening, hemoglobin fractionation, hemoglobin electrophoresis or DNA sequencing
- ☐ Pending – DO NOT ENTER DEMOGRAPHICS INTO DMS UNTIL CONFIRMED
- ☐ Unable to Confirm, subject not eligible – FORM COMPLETE

*******PATIENT SURVEY IS NOW ACCESSIBLE IN REDCap*******

<u>Subject Demographics for Confirmed Diagnoses Only:</u>

1. Date of birth — |___|___|/|___|___|/|___|___|___|___| (mm/dd/yyyy)

2. Race (check all that apply)
   - ☐ American Indian or Alaska Native
   - ☐ Asian
   - ☐ Black or African American
   - ☐ Native Hawaiian or Pacific Islander
   - ☐ White

3. Ethnicity (check one)
   - ☐ Hispanic or Latino
   - ☐ Not Hispanic or Latino

4. Sex
   - ☐ Male
   - ☐ Female

5. Zip code of primary residence — |___|___|___|___|___|

Unaffiliated patients have NOT been seen by a sickle cell provider (non-acute setting) as an outpatient in the past 2 years [for new patients, this excludes the visit during which they were enrolled].

6. Is this patient unaffiliated?
   - ☐ Yes
   - ☐ No

Medical Record Abstraction Form

Subject ID Label

If date or age is not available, enter '99'. Name of Abstractor: ____________________

1. DATE OF ENROLLMENT: |___|___|-|___|___|-|___|___|___|___|
2. Location where person enrolled:

- ☐ Routine visit--main SCDIC center
- ☐ Routine visit--satellite SCDIC center
- ☐ Emergency Department
- ☐ Acute Pain Center
- ☐ Hospital in-patient
- ☐ Primary Care offices
- ☐ Community event (e.g. SCD walk)
- ☐ Other ______________________________

3. Confirmed enrollment diagnosis: (CHECK ONLY ONE). DIAGNOSIS MUST BE SUPPORTED BY SOURCE DOCUMENTATION.

Diagnosis	
a. Hb SS or sickle cell anemia	☐
b. Hb SC disease	☐
c. Hb S beta^0 thalassemia	☐
d. Hb S beta$^+$ thalassemia	☐

Diagnosis	
e. Hb S hereditary persistence of fetal Hb (S/HPFH)	☐
f. Hb SE	☐
g. Hb SD	☐
h. Hb SO	☐

a. What was the basis for diagnosis?
- ☐ Newborn screening
- ☐ Hemoglobin fractionation
- ☐ Hemoglobin electrophoresis
- ☐ DNA sequencing

4. Approximate age of first diagnosis (physician confirmed): ________ AGE In YEARS **OR** ☐ NEWBORN SCREENING **OR** ☐ UNKNOWN

For subjects age 15-25 at time of enrollment:

a. Date of most recent visit to pediatric sickle cell provider. |__|__|-|__|__|-|__|__|__|__| ☐ DATE UNAVAILABLE
Date of first visit to adult sickle cell provider. |__|__|-|__|__|-|__|__|__|__| ☐ DATE UNAVAILABLE
☐ HAS NOT SEEN ADULT PROVIDER

☐ **FORM COMPLETE, MEDICAL RECORDS NOT AVAILABLE**

5. Ever tested for alpha-thalassemia?

- ☐ Yes—single alpha globin gene deleted
- ☐ Yes—two alpha globin genes deleted
- ☐ Yes––negative
- ☐ No—not evaluated
- ☐ Unknown

Basic Measurements (most recent)	Not in Record	Measurements	Date (mm/yyyy)	Steady state?
6. Height	☐	|___|___|___| CM		Y N
7. Weight	☐	|___|___|___|.|___| KG		Y N
8. Temperature	☐	|___|___|.|___| Celsius		Y N
9. Heart Rate	☐	|___|___|___| BEATS/MINUTE		Y N
10. Respiration Rate	☐	|___|___|___| BREATHS/MINUTE		Y N
11. Oxygen saturation (SpO_2)	☐	|___|___|___| %		Y N
12. Blood Pressure	☐	|___|___|___| / |___|___|___| ON ANTI-HYPERTENSIVE MEDS? ☐ Yes ☐ No		Y N

13. Has the subject ever used hydroxyurea? ☐ Yes
☐ No ➔ SKIP TO MEDICATION TABLE ON NEXT PAGE

a. Start date (mm/yyyy) ______/___________
b. Stop/last date (mm/yyyy) ______/___________
c. Total duration of use _____ ☐ Months or ☐ Years ☐ Unknown
d. Current dose _____ Mg/kg or _____ Mg

14. Please list all medications the subject is **currently** taking (at time of enrollment). ☐ NONE CURRENTLY BEING USED

Name of Medication	Name of Medication
a.	k.
b.	l.
c.	m.
d.	n.
e.	o.
f.	p.

Most recent visit to ….	Not in record	Visit/Admission Date (mm/yyyy)	Length of stay (in days)	Was visit for acute pain?	# of total visits in past year for acute pain/crisis
15. Acute Pain/Infusion Center (not admitted)	☐			☐ Yes ☐ No	
16. Emergency Department (not admitted)	☐			☐ Yes ☐ No	
17. Hospitalization	☐			☐ Yes ☐ No	

Most recent visit to…	Not in record	Visit Date (mm/yyyy)	Most recent visit to….	Not in record	Visit Date (mm/yyyy)
18. Primary care physician (i.e. family/internal medicine, pediatrician)	☐		19. Behavioral medicine/psychiatrist	☐	
20. Hematologist	☐		21. Orthopedic surgeon	☐	
22. Nephrologist	☐		23. Ophthalmologist	☐	
24. Cardiologist	☐		25. Neurologist	☐	
26. Pulmonologist	☐		27. OB/GYN	☐	

Transfusion History at Clinic Site

	None	# ever had	# total units	First time (mm/yyyy)	Last time (mm/yyyy)	Reason stopped	Frequency	Type
28. Episodic, simple	☐						☐ Less than once/year<br>☐ About once a year<br>☐ More than once/year<br>☐ Unknown	
29. Chronic, simple	☐					☐ Hemochromatosis<br>☐ Alloimmunization<br>☐ Other<br>☐ Unknown	☐ Once every 4 weeks<br>☐ Once every 6 weeks<br>☐ Once every 8 weeks<br>☐ Unknown	
30. Episodic, exchange	☐						☐ Less than once/year<br>☐ About once a year<br>☐ More than once/year<br>☐ Unknown	☐ Automated<br>☐ Manual<br>☐ Unknown
31. Chronic, exchange	☐					☐ Hemochromatosis<br>☐ Alloimmunization<br>☐ Other<br>☐ Unknown	☐ Once every 4 weeks<br>☐ Once every 6 weeks<br>☐ Once every 8 weeks<br>☐ Unknown	☐ Automated<br>☐ Manual<br>☐ Unknown

**SCD Complications**<br>Indicate whether the subject has <u>ever</u> had each condition and the date it was most recently diagnosed.	**No**	**Not in record**	**Yes**	**Most recent dx** (record age OR date)<br>**Age**	 **Date (mm/yyyy)**
**Musculoskeletal**					
32. Avascular necrosis *(check all that apply)*	☐	☐	☐		
a. Hip	☐	☐	☐		
b. Shoulder	☐	☐	☐		
c. Knee	☐	☐	☐		
33. Dactylitis	☐	☐	☐		
34. Osteomyelitis	☐	☐	☐		
**Genitourinary**					
35. Chronic kidney disease	☐	☐	☐		
36. End stage renal disease	☐	☐	☐		
a. Kidney transplant	☐	☐	☐		
37. Priapism	☐	☐	☐		
**Nervous system**					
38. Stroke (*check all that apply*)	☐	☐	☐		
a. Ischemic	☐	☐	☐		
b. Hemorrhagic	☐	☐	☐		
c. Transient ischemic attack (TIA)	☐	☐	☐		
d. Silent	☐	☐	☐		
39. Intracranial bleeding	☐	☐	☐		
**Cardiovascular**					
40. Pulmonary arterial hypertension	☐	☐	☐		
a. Mean pulmonary artery pressure > or = to 25 mm Hg	☐	☐	☐		
b. Tricuspid regurgitation velocity (TRV) > or = to 3.0 m/sec	☐	☐	☐		
41. Left ventricular dysfunction	☐	☐	☐		
**Respiratory**					
42. Acute chest syndrome	☐	☐	☐		
43. Asthma	☐	☐	☐		
**Digestive**					
44. Gallstones/cholelithiasis, cholecystitis	☐	☐	☐		
45. Splenomegaly *(check all that apply)*	☐	☐	☐		
a. Splenic sequestration	☐	☐	☐		
b. Splenic infarcts	☐	☐	☐		
c. Hypersplenism	☐	☐	☐		
d. Splenectomy	☐	☐	☐		
**Other Autoimmune/Inflammatory**					
46. Deep vein thrombosis (DVT)	☐	☐	☐		
a. Pulmonary embolism	☐	☐	☐		
b. Venous thromboembolism (VTE)	☐	☐	☐		
47. Lupus	☐	☐	☐		
48. Rheumatoid arthritis	☐	☐	☐		
49. Gout	☐	☐	☐		
50. Sarcoidosis	☐	☐	☐		
51. Other autoimmune or inflammatory, specify:	☐	☐	☐		

Other Conditions	No	Not in record	Yes	Most recent dx (record age OR date)	
				Age	Date (mm/yyyy)
52. Multi-organ failure *(check all that apply)*	☐	☐	☐		
a. ICU	☐	☐	☐		
b. Intubation	☐	☐	☐		
c. Simple transfusion	☐	☐	☐		
d. Exchange transfusion	☐	☐	☐		
e. Hemodialysis	☐	☐	☐		
f. Peritoneal dialysis	☐	☐	☐		
53. Pneumococcal sepsis (Pulmonary)	☐	☐	☐		
54. Skin ulcers (Integumentary)	☐	☐	☐		
55. Retinopathy (Ocular)	☐	☐	☐		
56. Diabetes mellitus (other systemic)	☐	☐	☐		
57. Iron overload (Other)	☐	☐	☐		
58. Chronic refractory pain (Other)	☐	☐	☐		
59. Anxiety (Mental health)	☐	☐	☐		
60. Depression (Mental health)	☐	☐	☐		
61. Other psychiatric disorder (Mental health) Specify:	☐	☐	☐		

62. Has the subject ever been diagnosed with cancer?

- ☐ Yes
- ☐ No ➔ GO TO Q 63
- ☐ Don't know ➔ GO TO Q 63

IF YES: For each primary cancer, complete a row in the table:

	Cancer Type & Location	Stage	When diagnosed? (record age or date)	
			Age	Date (mm/yyyy)
a.				
b.				

63. What kind of health insurance or health care coverage does the subject have at the time of enrollment? (Choose all that apply.)

- ☐ None
- ☐ Private health insurance
- ☐ Medicare
- ☐ Medicaid, Medical Assistance (MA), the Children’s Health Insurance Program (CHIP), or any kind of state or government-sponsored assistance.
- ☐ TRICARE or other military health care, including VA health care
- ☐ Other type of health insurance, specify:______________________

64. Year of first visit in medical record: |___|___|___|___| ☐ Subject not seen at this institution

PI review and sign-off: ________________________________

Laboratory Reporting Form

Subject ID Label

Complete using medical records, using values from the subject in steady state. **Abstractor**: ___________________

<table>
<tr><th>Test Name</th><th>Units</th><th>Date of Most Recent</th><th>NA</th></tr>
<tr><td>1. Nucleated RBC</td><td>|___|___|.|___| $10^3/mm^3$</td><td>_____/_____/_________</td><td>☐</td></tr>
<tr><td>2. White Blood Cells</td><td>|___|___|.|___| $10^3/mm^3$</td><td>_____/_____/_________</td><td>☐</td></tr>
<tr><td>3. RBC</td><td>|___|___|.|___| $10^6/mm^3$</td><td>_____/_____/_________</td><td>☐</td></tr>
<tr><td>4. Hemoglobin</td><td>|___|___|.|___|g/dL</td><td>_____/_____/_________</td><td>☐</td></tr>
<tr><td>5. Hematocrit</td><td>|___|___|.|___| %</td><td>_____/_____/_________</td><td>☐</td></tr>
<tr><td>6. MCV</td><td>|___|___| $micrometer^3$</td><td>_____/_____/_________</td><td>☐</td></tr>
<tr><td>7. MCH</td><td>|___|___|.|___| pg</td><td>_____/_____/_________</td><td>☐</td></tr>
<tr><td>8. MCHC</td><td>|___|___|.|___| g/dL</td><td>_____/_____/_________</td><td>☐</td></tr>
<tr><td>9. Platelets</td><td>|___|___|___|___| $10^3/mm^3$</td><td>_____/_____/_________</td><td>☐</td></tr>
<tr><td>10. Neutrophils (segmented and band together)</td><td>|___|___| %</td><td>_____/_____/_________</td><td>☐</td></tr>
<tr><td>11. Lymphocytes</td><td>|___|___| %</td><td>_____/_____/_________</td><td>☐</td></tr>
<tr><td>12. Monocytes</td><td>|___|___| %</td><td>_____/_____/_________</td><td>☐</td></tr>
<tr><td>13. Reticulocytes</td><td>|___|___|.|___| % AND/OR
|___|___| 10^3/microliter</td><td>_____/_____/_________</td><td>☐</td></tr>
<tr><td>14. Serum BUN</td><td>|___|___|.|___| mg/dL</td><td>_____/_____/_________</td><td>☐</td></tr>
<tr><td>15. Serum Creatinine</td><td>|___|.|___| mg/dL</td><td>_____/_____/_________</td><td>☐</td></tr>
<tr><td>17. Estimated creatinine clearance</td><td>|___|___|___|___| mL/min</td><td>_____/_____/_________</td><td>☐</td></tr>
<tr><td>18. Total Cholesterol</td><td>|___|___|___|___| mg/dL</td><td>_____/_____/_________</td><td>☐</td></tr>
<tr><td>19. Non-Fasting HDL</td><td>|___|___|___|___| mg/dL</td><td>_____/_____/_________</td><td>☐</td></tr>
<tr><td>20. Fasting HDL</td><td>|___|___|___|___| mg/dL</td><td>_____/_____/_________</td><td>☐</td></tr>
<tr><td>21. Non-Fasting LDL</td><td>|___|___|___|___| mg/dL</td><td>_____/_____/_________</td><td>☐</td></tr>
<tr><td>22. Fasting LDL</td><td>|___|___|___|___| mg/dL</td><td>_____/_____/_________</td><td>☐</td></tr>
<tr><td>23. Triglyceride</td><td>|___|___|___|___| mg/dL</td><td>_____/_____/_________</td><td>☐</td></tr>
<tr><td>24. Non-Fasting Blood Glucose</td><td>|___|___|___|___| mg/dL</td><td>_____/_____/_________</td><td>☐</td></tr>
<tr><td>25. Fasting Blood Glucose</td><td>|___|___|___|___| mg/dL</td><td>_____/_____/_________</td><td>☐</td></tr>
<tr><td>26. CRP</td><td>|___|___|.|___| mg/dL</td><td>_____/_____/_________</td><td>☐</td></tr>
<tr><td>27. Bilirubin serum, total</td><td>|___|___|.|___| mg/dL</td><td>_____/_____/_________</td><td>☐</td></tr>
<tr><td>28. Bilirubin, serum, direct</td><td>|___|___|.|___| mg/dL</td><td>_____/_____/_________</td><td>☐</td></tr>
<tr><td>29. AST</td><td>|___|___|.|___| U/L</td><td>_____/_____/_________</td><td>☐</td></tr>
</table>

<table>
<tr><th>Test Name</th><th>Units</th><th>Date of Most Recent</th><th>NA</th></tr>
<tr><td>30. ALT</td><td>|___|___|.|___| U/L</td><td>_____/_____/_________</td><td>☐</td></tr>
<tr><td>31. Alkaline Phosphatase</td><td>|___|___|___|.|___| U/L</td><td>_____/_____/_________</td><td>☐</td></tr>
<tr><td>32. Total Protein (plasma)</td><td>|___|___|.|___| g/dL</td><td>_____/_____/_________</td><td>☐</td></tr>
<tr><td>33. Albumin</td><td>|___|___|.|___| g/dL</td><td>_____/_____/_________</td><td>☐</td></tr>
<tr><td>34. LDH (serum)</td><td>|___|___|___| U/L</td><td>_____/_____/_________</td><td>☐</td></tr>
<tr><td>35. NT-pro-BNP</td><td>|___|___|___|___| pg/mL</td><td>_____/_____/_________</td><td>☐</td></tr>
<tr><td>36. BNP</td><td>|___|___|___|___| pg/mL</td><td>_____/_____/_________</td><td>☐</td></tr>
<tr><td>37. Serum iron</td><td>|___|___|___| ug/dL</td><td>_____/_____/_________</td><td>☐</td></tr>
<tr><td>38. Total iron binding capacity (TIBC)</td><td>|___|___|___| ug/dL</td><td>_____/_____/_________</td><td>☐</td></tr>
<tr><td>39. Serum transferrin</td><td>|___|___|___| mg/dL</td><td>_____/_____/_________</td><td>☐</td></tr>
<tr><td>40. Ferritin</td><td>|___|___|___| ng/mL</td><td>_____/_____/_________</td><td>☐</td></tr>
<tr><td>41. 25-Hydroxy Vitamin D</td><td>|___|___| ng/mL</td><td>_____/_____/_________</td><td>☐</td></tr>
<tr><td>42. Erythropoietin (EPO)</td><td>|___|___| mU/ml</td><td>_____/_____/_________</td><td>☐</td></tr>
<tr><td>43. Urine albumin</td><td>|___|___|___|.|___| mg/g</td><td>_____/_____/_________</td><td>☐</td></tr>
<tr><td>44. Urine albumin / creatinine</td><td>|___|.|___|___| mcg/mg</td><td>_____/_____/_________</td><td>☐</td></tr>
<tr><td>45. Urine protein (dipstick)</td><td>|__| 0/negative |__| trace
|__| 1+ |__| 2+ |__| 3+ |__| 4+ |__| positive</td><td>_____/_____/_________</td><td>☐</td></tr>
<tr><td>46. Urine protein/creatinine</td><td>|___|.|___|___| mg/g</td><td>_____/_____/_________</td><td>☐</td></tr>
<tr><td>47. Urine dipstick heme</td><td>|__| 0/negative |__| trace
|__| 1+ |__| 2+ |__| 3+ |__| 4+ |__| positive</td><td>_____/_____/_________</td><td>☐</td></tr>
<tr><td>48. Urine microscopic RBCs</td><td>|___|___|.|___| $10^3/mm^3$ (if < 100, enter exact value) OR |__| $\geq$ 100 $10^3/mm^3$</td><td>_____/_____/_________</td><td>☐</td></tr>
<tr><td>49. Urine microscopic WBCs</td><td>|___|___|.|___| $10^3/mm^3$ (if < 100, enter exact value) OR |__| $\geq$ 100 $10^3/mm^3$</td><td>_____/_____/_________</td><td>☐</td></tr>
<tr><td>50. Hemoglobin fractionation, baseline (before HU use)</td><td>Hb A |___|___|% Hb A2 |___|___|%
Hb C |___|___|% Hb D |___|___|%
Hb E |___|___|% Hb F |___|___|%
Hb O |___|___|% Hb S |___|___|%
Other, _________ |___|___|%</td><td>_____/_____/_________</td><td>☐</td></tr>
<tr><td>51. Hemoglobin fractionation, most recent</td><td>Hb A |___|___|% Hb A2 |___|___|%
Hb C |___|___|% Hb D |___|___|%
Hb E |___|___|% Hb F |___|___|%
Hb O |___|___|% Hb S |___|___|%
Other, _________ |___|___|%</td><td>_____/_____/_________</td><td>☐</td></tr>
<tr><td>52. Hemoglobin fractionation, maximum dose HU</td><td>Hb A |___|___|% Hb A2 |___|___|%
Hb C |___|___|% Hb D |___|___|%
Hb E |___|___|% Hb F |___|___|%
Hb O |___|___|% Hb S |___|___|%
Other, _________ |___|___|%</td><td>_____/_____/_________</td><td>☐</td></tr>
</table>

Renal Form

Subject ID Label

DATE FORM COMPLETED: |___|___|-|___|___|-|___|___|___|___|

This form should be completed if YES to either Q35 (chronic kidney disease) or Q36 (end stage renal disease) on the enrollment Medical Record Abstraction Form.

	**1. Albuminuria**	**2. Proteinuria**
**a. When did it start?**	|__|__|-|__|__|-|__|__|__|__| **OR**<br>☐ Less than 1 year ago<br>☐ Between 1 and 2 years ago<br>☐ More than 2 years ago<br>☐ Unknown/NA<br>☐ Has not had albuminuria **GO TO Q2**	|__|__|-|__|__|-|__|__|__|__| **OR**<br>☐ Less than 1 year ago<br>☐ Between 1 and 2 years ago<br>☐ More than 2 years ago<br>☐ Unknown/NA<br>☐ Has not had proteinuria **GO TO Q3**
**b. Date of most recent measurement**	|__|__|-|__|__|-|__|__|__|__| ☐ Unknown/NA<br>☐ No measurement available **GO TO Q2**	|__|__|-|__|__|-|__|__|__|__| ☐ Unknown/NA<br>☐ No measurement available **GO TO Q3**
**c. Type of measurement *(check one)***	☐ Spot<br>☐ 24-hour urine **GO TO Q2**<br>☐ Unknown/NA **GO TO Q2**	☐ Spot<br>☐ 24-hour urine **GO TO Q3**<br>☐ Unknown/NA **GO TO Q3**
**d. Spot urine sample**	_______ mg/L (milligram albumin per liter of urine)	_______ mg/L (milligram protein per liter of urine)
**e. Spot urine [albumin/protein]/ creatinine ratio**	_______ mg/mmol (milligram albumin per millimole creatinine)<br>_______ µg/mg (microgram albumin per milligram creatinine)	_______ mg/mmol (milligram protein per millimole creatinine)<br>_______ µg/mg (microgram protein per milligram creatinine)

	**3. eGFR <60**
**a. When did it start?**	|__|__|-|__|__|-|__|__|__|__| **OR**<br>☐ Less than 1 year ago<br>☐ Between 1 and 2 years ago<br>☐ More than 2 years ago<br>☐ Unknown/NA<br>☐ Has not had albuminuria **GO TO Q4**
**b. Date of most recent measurement**	|__|__|-|__|__|-|__|__|__|__| ☐ Unknown/NA<br>☐ No measurement available **GO TO Q4**

**4. Has the subject had:**	**Yes**	**No**	**Unknown**
a. History of acute kidney injury (AKI*)	☐	☐	☐
b. History of >1 episode of AKI*	☐	☐	☐
c. Hemodialysis	☐	☐	☐
d. Peritoneal dialysis	☐	☐	☐
e. Kidney disease/ESRD	☐<br><br>Date: |_|_|-|_|_|-|_|_|_|_| **OR**<br>☐ Less than 1 year ago<br>☐ Between 1 and 2 years ago<br>☐ More than 2 years ago<br>☐ Unknown/NA	☐	☐
f. Kidney transplant	☐<br><br>Date: |_|_|-|_|_|-|_|_|_|_| ☐ Unknown/NA<br><br>Rejection?<br>☐ Yes<br>☐ No<br>☐ Unknown/NA	☐	☐

*Must meet Acute Kidney Injury Network (AKIN) criteria, with a minimum of stage 1: an increase in serum creatinine of ≥26.4 µmol/L or increase to ≥150–200% from baseline.

Pulmonary hypertension and LV dysfunction Form

Subject ID Label

DATE FORM COMPLETED: |___|___|-|___|___|-|___|___|___|___|

Complete this form if there is a YES response to Q40. pulmonary hypertension OR Q41. LV dysfunction on the Enrollment Medical Record Abstraction Form. Use the most recent results available in the 5 years prior to the date of consent for the Registry.

Date of most recent ECHO: |___|___|-|___|___|-|___|___|___|___| ☐ ECHO not available

Measurement from ECHO		Qualitative data	Quantitative data
1.	Mitral regurgitation	☐ none<br>☐ trivial ☐ moderate<br>☐ mild ☐ severe	
2.	Tricuspid regurgitation	☐ none<br>☐ trivial ☐ moderate<br>☐ mild ☐ severe	
3.	TR jet velocity		_____m/s
4.	Tricuspid Annular Plane Systolic Excursion		________mm
5.	Ejection fraction, left ventricle		|__|__|.|__| %
6.	Left Atrial Volume	☐ normal ☐ moderate<br>☐ mild ☐ severe	(LAESVI in ml/sq.m) = _______ml/m²
7.	Right Atrial Volume	☐ normal ☐ moderate<br>☐ mild ☐ severe	(RAESVI in ml/sq.m) = _______ml/m²
8.	Left ventricular volume	☐ normal ☐ moderate<br>☐ mild ☐ severe	Left ventricular end systolic dimension<br>LVIDs =________mm<br>LVESVI= ____ mL/m2<br><br>Left ventricular end diastolic dimension<br>LVIDd=_________mm<br><br>Left ventricular posterior wall mm thickness at end-diastole<br>LVPwD=_______mm
9.	Right ventricular volume	☐ normal ☐ moderate<br>☐ mild ☐ severe	Right ventricular end systolic dimension<br>RVIDs=________mm<br><br>Right ventricular end diastolic dimension<br>RVIDd=_________mm<br>Mention of interventricular septal flattening Y/N
10.	RV hypertrophy	☐ normal ☐ moderate<br>☐ mild ☐ severe	

Date of most recent EKG: |___|___|-|___|___|-|___|___|___|___| ☐ EKG not available

Measurement from EKG		Diagnosed?
11.	Arrhythmia	☐ Yes ➔ type ____________<br>☐ No<br>☐ Unknown
12.	Ventricular rate	________ bpm ☐ NA
13.	PR Interval	________ ms ☐ NA
14.	QRS duration	________ ms ☐ NA
15.	QT/QTc	____ /____ ms ☐ NA
16.	P-R-T axes	____ ____ ____ ☐ NA

Date of most recent right heart catheterization: |___|___|-|___|___|-|___|___|___|___| ☐ Report not available

Target		Measurement
17.	RA pressure (mean)	________ mm/hg ☐ NA
18.	RV pressure (mean)	________ mm/hg ☐ NA
19.	PA pressure (mean)	________ mm/hg ☐ NA
20.	Pulmonary artery saturation	________ % ☐ NA
21.	Pulmonary vascular resistance	________ dynes-sec-cm^{-5} ☐ NA
22.	Pulmonary capillary wedge pressure (PCWP or PAWP)	________ mm/hg ☐ NA
23.	Cardiac output and index	________ L/min ☐ NA

Notes:

1) Right atrial pressure: This is usually present in the echo report and is reported based on IVC collapsibility (might be under heading of IVC/Hepatic veins)

2) Right atrial size: qualitatively (as normal, mildly, moderately or severely dilated) vs. quantitatively (RA area or RAESVI). The numerical values are all usually reported at the bottom of the report.

3) Left ventricular size (qualitative - normal, mild, mod, severely dilated) vs quantitative (LVEDVI , LVESVI)

4) Any comment of **interventricular septal flattening** indicates RV pressure or volume overloading and points to significant pulmonary hypertension.

5) Left atrial dimensions reported qualitatively (as normal, mildly, moderately or severely dilated) vs. quantitatively (LAESVI in ml/sq.m). The numerical values are all usually reported at the bottom of the report.

Off Study Form

Subject ID Label

Complete this form to document significant off-study events that have occurred to enrolled subjects. By reporting a significant event, the subject will not be considered for any scheduled follow-up activities.

1. Event
   - ☐ Ineligible (data will be destroyed) → **STOP**
   - ☐ Duplicate enrollment, delete/merge data for this ID number → **STOP**
   - ☐ Withdrew from study, Reason: ____ → **GO TO Q2**
   - ☐ Loss to follow-up, Reason: ____ → **GO TO Q3**
   - ☐ Death → **GO TO Q4**

2. FOR WITHDRAWLS: Do data need to be destroyed? ☐ Yes ☐ No → **STOP**

3. Date last known alive |___|___|-|___|___|-|___|___|___|___| → **STOP, complete Follow-up Form up to this date**
   Month Day Year

4. Date of death |___|___|-|___|___|-|___|___|___|___| → **CONTINUE, complete Follow-up Form up to this date**
   Month Day Year

5. Cause of Death on death certificate
   Primary cause: ____
   Secondary cause(s): ____

   ☐ Death certificate Not Available

6. Other sources of Cause of Death (check **all** that apply)

   ☐ No Other Sources ☐ Family Member ☐ Medical Record ☐ Autopsy Report

   a. Cause of Death from other sources (identify in consultation with PI)

	Primary/ Immediate Cause (check one)	Secondary/ Underlying or Comorbid Causes (check all that apply)
Acute Chest Syndrome	☐	☐
Respiratory Failure	☐	☐
Sudden Death	☐	☐
Infection	☐	☐
Stroke, Ischemic	☐	☐
Stroke, Hemorrhagic	☐	☐
Cardiac Arrest	☐	☐
Sickle Cell Disease Multiorgan Failure Syndrome	☐	☐
Kidney Failure	☐	☐
Liver Failure	☐	☐
Pulmonary Embolism	☐	☐
Trauma	☐	☐
Cancer (specify type, location): ____________________	☐	☐
Other primary (specify): __________________________	☐	☐
Other secondary (specify): ________________________	☐	☐

7. Is an autopsy report available? (copies of autopsy reports should be maintained locally) ☐ Yes ☐ No

Subject ID

SCDIC

Sickle Cell Disease Implementation Consortium

Patient Follow-up Survey

Final Version 1.0, 2/18/2019

INSTRUCTION:
We are interested in learning how you have been doing since we were last in touch with you. As you complete this form, answer the questions as best as you can. If you don't know the answer or do not want to answer a question, you may leave it blank.

What is today's date? |__|__|- |__|__|- |___|___|___|___|
Month Day Year

A. YOUR RECENT PAIN

1. Do you take pain medicine every day for your sickle cell disease? ❐ Yes ❐ No

2. What pain medicines do you currently take for your sickle cell disease? On the list below, check the box next to the name of the **pain medicines** you take (even if not everyday). Check here if you don't take pain medicines → ❐

PAIN MEDICATIONS	
❐ acetaminophen & codeine (Tylenol-Codeine #3 or #4)	❐ magnesium salicylic acid (Durasal)
❐ acetaminophen & oxycodone (Percocet, Endocet)	❐ meperidine (Demerol)
❐ acetaminophen & hydrocodone (Vicodin, Norco, Lortab)	❐ methadone (Dolophine)
❐ acetaminophen (Tylenol)	❐ morphine sulfate (MS Contin, Kadian)
❐ amitriptyline/Elavil	❐ morphine and naltrexone (Embeda, MS IR)
❐ aspirin (any brand)	❐ naproxen (Aleve, Naprosyn)
❐ buprenorphine/Belbuca/Butrans	❐ oxycodone (Oxycontin, Roxicodone)
❐ butalbital, acetaminophen, and caffeine (Fioricet)	❐ oxymorphone (Opana)
❐ butalbital, aspirin, caffeine, & codeine (Ascomp-Codeine)	❐ pentazocine/Talwin
❐ celecoxib (Celebrex)	❐ pregabalin (Lyrica)
❐ diclofenac/Voltaren/Cambia/Solaraze	❐ promethazine/Phenergan with codeine
❐ esomeprazole (Nexium)	❐ tapentadol/Nucynta
❐ Excedrin	❐ tramadol
❐ fentanyl (Duragesic)	❐ venlafaxine/Effexor
❐ gabapentin (Neurontin)	❐ medical marijuana/cannabis
❐ hydromorphone (Exalgo ER, Dilaudid)	❐ topical/skin cream for pain (all types)
❐ ibuprofen (Motrin, Advil)	❐ Other pain medication (specify below)
❐ ketorolac/Toradol	

3. In the past 12 months, how many sickle cell pain attacks (crises) did you have?
   - ❐ I did not have a pain attack in the past 12 months
   - ❐ 1
   - ❐ 2
   - ❐ 3
   - ❐ 4 or more

4. When was your last pain attack (crisis)?
   - ❐ I've never had a pain attack (crisis) → **skip to Question 8**
   - ❐ More than a year ago → **skip to Question 8**
   - ❐ 7-11 months ago
   - ❐ 1-6 months ago
   - ❐ 1-3 weeks ago
   - ❐ Less than a week ago
   - ❐ I have one right now

5. How severe was your pain during your last pain attack (crisis)? **Circle a number from 0 to 10 below**, where 0 is no pain and 10 is the worst pain imaginable.

No Pain	0	1	2	3	4	5	6	7	8	9	10	Worst Pain Imaginable

6. How much did your last pain attack (crisis) interfere with your life?

- ❒ Not at all, I did everything I usually do
- ❒ I had to cut down on some things I usually do
- ❒ I could not do most things I usually do
- ❒ I could not take care of myself and needed some help from family or friends
- ❒ I could not take care of myself and needed constant care from family, friends, doctors, or nurses

7. About how long did your most recent pain attack (crisis) last?

- ❒ Less than 1 hour
- ❒ 1-12 hours
- ❒ 13-23 hours
- ❒ 1-3 days
- ❒ 4-6 days
- ❒ 1-2 weeks
- ❒ More than 2 weeks

8. Think about your pain in the **past 7 days**, and answer the following questions.

		Never	Rarely	Sometimes	Often	Always
a.	How often did you have very severe pain?	❒	❒	❒	❒	❒
b.	How often did you have pain so bad that it was hard to finish what you were doing?	❒	❒	❒	❒	❒

9. Now think about your pain in the **past 6 months**, and answer the following questions.

		Never	Rarely	Sometimes	Often	Always
a.	How often did you have very severe pain?	❒	❒	❒	❒	❒
b.	How often did you have pain so bad that it was hard to finish what you were doing?	❒	❒	❒	❒	❒

10. Think about how your pain felt in the **past 7 days**, and answer the following questions.

		Not at all	A little bit	Somewhat	Quite a bit	Very much
a.	Did your pain feel like pins and needles?	❒	❒	❒	❒	❒
b.	Did your pain feel sore?	❒	❒	❒	❒	❒

11. Would you say that your pain management plan is…..

- ❒ Effective for managing your pain
- ❒ Somewhat effective for managing your pain
- ❒ Ineffective at managing your pain
- ❒ You don’t have a pain management plan

B. YOUR MEDICAL CONDITIONS

12. Do you get regular blood transfusions for your sickle cell disease?

- ❒ Yes
- ❒ No

13. In the past 12 months, how many units (pints) of blood have you received?

- ❒ None
- ❒ 1 – 2
- ❒ 3 – 5
- ❒ 6 – 10
- ❒ 11 – 15
- ❒ >15
- ❒ Don't Know

14. Are you **currently** on iron chelation treatment (e.g., Desferal, Exjade, Jadenu, deferasirox, Ferriprox, deferiprone, phlebotomy)?

- ❒ Yes
- ❒ No
- ❒ Don't Know

15. In the past 12 months, has your spleen been removed?

- ❒ Yes
- ❒ No

16. In the past 12 months, have you been **newly diagnosed** with any of the following conditions?

	Condition	YES	NO
a.	Lung problems such as pneumonia or acute chest syndrome	❒	❒
b.	Kidney damage	❒	❒
c.	Eye damage called retinopathy	❒	❒
d.	Damage to your hip or shoulder due to sickle cell disease	❒	❒
e.	High blood pressure in your lungs (also called pulmonary hypertension)	❒	❒
f.	Heart failure	❒	❒
g.	Blood clots in your legs or arms or that went to your lung	❒	❒
h.	A stroke	❒	❒
i.	Asthma	❒	❒
j.	Diabetes	❒	❒
k.	Liver problems such as hepatitis, iron overload, or cirrhosis	❒	❒
l.	Skin ulcers	❒	❒

C. HYDROXYUREA USE

17. In the past 12 months, have you taken hydroxyurea?

- ❒ Yes
- ❒ No → ***skip to Section D***

18. Are you **currently** taking hydroxyurea?

- ❒ Yes → ***skip to Question 20***
- ❒ No

19. In the past 12 months, what is the reason you discontinued or stopped taking hydroxyurea? Please select one from the list below.

- ❒ Side effects
- ❒ Personal preference
- ❒ Provider decision
- ❒ Didn't work
- ❒ Pregnancy concerns
- ❒ Other reason not listed above, specify________________________________

20. How many days did you take hydroxyurea in the PAST WEEK?

❒ 0 days ❒ 1 day ❒ 2 days ❒ 3 days ❒ 4 days ❒ 5 days ❒ 6 days ❒ 7 days

21. In the last 12 months, which of the following side effects did you experience while you were taking hydroxyurea? Select one or more from the list below.

❒ Hair loss/thinning
❒ Nail blackening or discoloration
❒ Lowered blood counts (e.g., platelets, white count, hemoglobin)
❒ Low sperm count or other fertility problems
❒ Nausea/vomiting
❒ Skin ulcers
❒ Weight gain
❒ Headaches or dizziness
❒ Fatigue/drowsiness
❒ No side effects

22. In the last 12 months, what makes it difficult for you to take hydroxyurea, or is there a reason why you do not take hydroxyurea? Select one or more from the list below, whether or not you have ever taken hydroxyurea.

❒ I have no difficulties or concerns using hydroxyurea
❒ I don't know enough about the medicine
❒ Sometimes I forget to take the medicine
❒ I am worried about side effects
❒ I don't like the frequent blood tests or clinic visits
❒ I'm feeling well and I don't think I need it
❒ The cost is more than I can afford
❑ I have heard that hydroxyurea may cause cancer
❑ I have heard that hydroxyurea may cause problems with having healthy children
❒ Other difficulty, specify___________________________

D. OTHER MEDICATIONS YOU ARE TAKING

23. In the past 12 months, have you taken the drug called Endari (l-glutamine)?

❒ Yes
❒ No → ***skip to Question 28***

24. Are you **currently** taking Endari?

❒ Yes → ***skip to Question 26***
❒ No

25. In the past 12 months, what is the reason you discontinued or stopped taking Endari? Please select one from the list below.

❒ Side effects
❒ Personal preference
❒ Provider decision
❒ Didn't work
❒ Other reason <u>not listed above</u>, specify_______________________________

26. How many days did you take Endari in the PAST WEEK?

❒ 0 days ❒ 1 day ❒ 2 days ❒ 3 days ❒ 4 days ❒ 5 days ❒ 6 days ❒ 7 days

27. In the last 12 months, what side effects have you experienced while you were taking Endari? Select one or more from the list below.

- ❒ No side effects
- ❒ Nausea/vomiting
- ❒ Stomach pain
- ❒ Cough
- ❒ Headaches or dizziness
- ❒ Other not listed above ____

28. We would like to know what other types of medications you are **currently** taking, ***excluding pain medications, iron chelators, hydroxyurea, and Endari*** which we already asked about.

Review the list in the table below and check the box next to the type of medications you are **currently** taking.

**CATEGORIES OR TYPES OF DRUGS YOU MAY BE TAKING**	
❒ Allergy drugs	❒ High cholesterol drugs
❒ Asthma or COPD inhalers (bronchodilators)	❒ Hypothyroid drugs
❒ Antibiotics	❒ Insomnia drugs & sleep aides
❒ ADD/ADHD drugs	❒ Nausea drugs
❒ Anti-seizure drugs	❒ Muscle relaxers
❒ Anti-anxiety drugs	❒ Stool softeners and laxatives
❒ Antidepressants	**TYPES OF VITAMINS**
❒ Birth control	❒ Iron supplements
❒ Blood thinning drugs (anticoagulants)	❒ Folic acid
❒ Diabetes drugs	❒ Vitamin D, all types
❒ Diuretics, fluid/water retention pills	❒ Multi-vitamins
❒ Heartburn, indigestion, acid reflux drugs	❒ Any other vitamins and supplements

29. Are you taking any medications for **high blood pressure** (hypertension) or for your **heart**? ❒ Yes ❒ No

If yes, what is the name of the high blood pressure or heart medication? ________________________________

30. Are you taking any <u>other type</u> of medication that we did not already ask about? ❒ Yes ❒ No

If yes, what is the name of the other medication(s)? ____

31. Are you currently participating in a study where you are taking a medicine for sickle cell? ❒ Yes ❒ No

E. YOUR SLEEP

32. Think about your sleep in the **past 7 days**, and answer the following questions.

		Never	Rarely	Sometimes	Often	Always
a.	How often did you stay up most of the night because you could not fall asleep?	❒	❒	❒	❒	❒
b.	How often did you have a lot of trouble falling asleep?	❒	❒	❒	❒	❒

F. YOUR SOCIAL AND MENTAL HEALTH

33. Think about how you felt in the **past 7 days**, and respond to each question or statement.

		Never	Rarely	Sometimes	Often	Always
a.	I felt worthless.	❒	❒	❒	❒	❒
b.	I felt helpless.	❒	❒	❒	❒	❒
c.	I felt depressed.	❒	❒	❒	❒	❒
d.	I felt hopeless.	❒	❒	❒	❒	❒
e.	How often did you feel completely hopeless because of your health?	❒	❒	❒	❒	❒
f.	How often were you very worried about needing to go to the hospital?	❒	❒	❒	❒	❒
		**Not at all**	**A little bit**	**Somewhat**	**Quite a bit**	**Very much**
g.	I felt tired.	❒	❒	❒	❒	❒

34. In the **past 7 days**, how often did the following happen?

		Never	Rarely (Once)	Sometimes (2-3 times)	Often (once a day)	Very often (several times a day)
a.	I had to read something several times to understand it.	❒	❒	❒	❒	❒
b.	My thinking was slow.	❒	❒	❒	❒	❒
c.	I had to work really hard to pay attention or I would make a mistake.	❒	❒	❒	❒	❒
d.	I had trouble concentrating.	❒	❒	❒	❒	❒

35. How much DIFFICULTY do you **currently** have doing the following things?

		None	A little	Somewhat	A lot	Cannot do
a.	Reading and following complex instructions (e.g., directions for a new medication)?	❒	❒	❒	❒	❒
b.	Planning for and keeping appointments that are not part of your weekly routine (e.g., therapy or doctor appointment, social gathering with friends/family)?	❒	❒	❒	❒	❒
c.	Managing your time to do most of your daily activities?	❒	❒	❒	❒	❒
d.	Learning new tasks or instructions?	❒	❒	❒	❒	❒

36. In the **past 30 days**, how much did the following happen?

		Not at all	A little bit	Somewhat	Quite a bit	Very much
a.	How much did you rely on others to take care of you because of your health?	❒	❒	❒	❒	❒
b.	How much did your health make it hard for you to do things with your friends?	❒	❒	❒	❒	❒

G. YOUR ABILITY TO MANAGE YOUR SICKLE CELL DISEASE

37. How often do you need to have someone help you when you read instructions, pamphlets, or other written material from your doctor or pharmacy?

- ❒ Never
- ❒ Rarely
- ❒ Sometimes
- ❒ Often
- ❒ Always

38. Please respond to each statement below by marking one box per row.

CURRENT Level of Confidence (confidence is how sure you are about each statement)		I am not at all confident	I am a little confident	I am somewhat confident	I am quite confident	I am very confident
a.	I can follow directions when my doctor changes my medications.	❒	❒	❒	❒	❒
b.	I can take my medication when there is a change in my usual day (unexpected things happen).	❒	❒	❒	❒	❒
c.	I can manage my medication without help.	❒	❒	❒	❒	❒
d.	I can list my medications, including the doses and schedule.	❒	❒	❒	❒	❒

H. BARRIERS TO YOUR MEDICAL CARE

39. During the past 12 months, was there any time when you didn't get the medical care you needed or had delays in getting the care you needed?

- ❒ Yes
- ❒ No → ***skip to END***

40. In the past 12 months, did you not get the medical care you needed or have delays getting medical care you needed for any of the following reasons? Select one or more from the list below.

- ❒ Worry about the cost
- ❒ The doctor or hospital wouldn‘t accept your health insurance
- ❒ Your health plan wouldn‘t pay for the treatment
- ❒ You couldn‘t get an appointment soon enough
- ❒ You couldn‘t get there when the doctor‘s office or clinic was open
- ❒ It takes too long to get to the doctor‘s office or clinic from your house or work
- ❒ You couldn‘t get through on the telephone
- ❒ You were too busy with work or other commitments to take the time
- ❒ You didn‘t think the problem was serious enough
- ❒ You had previous bad experiences with the health care system
- ❒ People at the doctor’s office or clinic don’t speak the same language I do
- ❒ Some other reason not listed above, please specify ____

This is the END of the survey. Please return it to the study coordinator. Thank you!

Adverse Childhood Experience Questionnaire (ACE-Q)

Version 1.0 (2/18/2019)

Subject ID

Today's date: |__|__|/ |__|__|/ |_2_|_0_|___|___|
Month Day Year

This form asks questions about events that happened during your childhood. Please read the statements below. Count the number of statements that apply to you and write the total number in the box provided. **Please DO NOT mark or indicate which specific statements apply to you.**

1) Of the statements in Section 1, HOW MANY apply to you? Write the total number in the box: ☐

Section 1. *At any point before you were age 18:*

- Your parents or guardians were separated or divorced
- You lived with a household member who served time in jail or prison
- You lived with a household member who was depressed, mentally ill or attempted suicide
- You saw or heard household members hurt or threaten to hurt each other
- A household member swore at, insulted, humiliated, or put you down in a way that scared you OR a household member acted in a way that made you afraid that you might be physically hurt
- Someone touched your private parts or asked you to touch their private parts in a sexual way that was unwanted, against your will, or made you feel uncomfortable
- More than once, you went without food, clothing, a place to live or had no one to protect you
- Someone pushed, grabbed, slapped or threw something at you OR you were hit so hard that you were injured or had marks
- You lived with someone who had a problem with drinking or using drugs
- You felt unsupported, unloved and/or unprotected

2) Of the statements in Section 2, HOW MANY apply to you? Write the total number in the box: ☐

Section 2. *At any point before you were age 18:*

- You were in foster care
- You experienced harassment or bullying at school
- You lived with a parent or guardian who died
- You were separated from your primary caregiver through deportation or immigration
- You had a serious medical procedure or life threatening illness
- You often saw or heard violence in the neighborhood or in your school neighborhood
- You were detained, arrested or incarcerated
- You were often treated badly because of race, sexual orientation, place of birth, disability or religion
- You experienced verbal or physical abuse or threats from a romantic partner (i.e., boyfriend or girlfriend)

This is the END of the survey. Please return it to the study coordinator.

Thank you for your participation.
